# Programmable DNA Folding Modulates Phase Behavior and Dynamics of DNA/Peptide Condensates

**DOI:** 10.1021/acsnano.6c03646

**Published:** 2026-05-13

**Authors:** Itai Katzir, Yanbing Wen, Inbal Razi, Vadim Skoi, Roy Beck, Hao Dong, Ayala Lampel

**Affiliations:** † Shmunis School of Biomedicine and Cancer Research, George S. Wise Faculty of Life Sciences, 26745 Tel Aviv University, Tel Aviv 6997801, Israel; ‡ State Key Laboratory of Analytical Chemistry for Life Science, Kuang Yaming Honors School, Chemistry and Biomedicine Innovation Center (Chem-BIC), ChemBioMed Interdisciplinary Research Center at Nanjing University, Institute for Brain Sciences, 12581 Nanjing University, Nanjing 210023, China; § School of Physics and Astronomy, Tel Aviv University, Tel Aviv 6997801, Israel; ∥ Center for the Physics and Chemistry of Living Systems, Tel Aviv University, Tel Aviv 6997801, Israel; ⊥ Center for Nanoscience and Nanotechnology, Tel Aviv University, Tel Aviv 6997801, Israel; # Sagol School of Neuroscience, Tel Aviv University, Tel Aviv 6997801, Israel; ∇ Division Polymer Biomaterials Science, Leibniz Institute of Polymer Research Dresden, 01069 Dresden, Germany

**Keywords:** Liquid−liquid phase separation (LLPS), Biomolecular condensates, Coacervates, Peptide, DNA

## Abstract

Membraneless compartments formed by liquid–liquid phase separation (LLPS) regulate biochemical reactions and play a key role in both physiological and pathological processes, including viral replication. In retroviral systems, the extent of genome folding is critical for the efficient packaging of new viral particles, a process mediated by the nucleocapsid (NC) protein that chaperones RNA folding and assembly. Here, we sought to elucidate how nucleic-acid folding and structural folding influence LLPS and whether an HIV NC-derived peptide (HNP) can modulate this process through chaperone-like activity. To this end, we designed a programmable single-stranded DNA (ssDNA) library spanning varying degrees of folding and palindromic architectures, enabling systematic investigation of how nanoscale structural order governs coacervation. Using circular dichroism, FRET, SAXS, and coarse-grained simulations, we correlate DNA conformations with phase behavior and emergent condensate material properties. We find that interactions with HNP promote DNA folding and that increasing DNA order suppresses LLPS, whereas structural disorder and palindromic linkers that induce DNA dimerization enhance phase separation by facilitating multivalent interactions and in turn increasing condensate viscosity. Together, these findings identify two programmable determinants, local structural order and palindromic dimerization, that govern DNA/peptide condensate behavior, offering mechanistic insight into viral genome organization and guiding principles for tuning the physicochemical and material properties of synthetic condensates.

## Graphical abstract

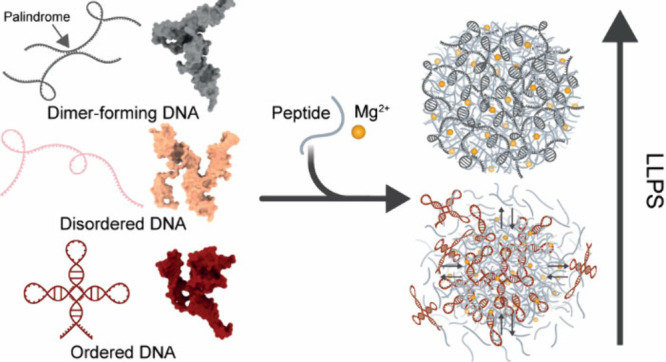

## Introduction

Membraneless compartments formed by liquid–liquid phase separation (LLPS) spatially organize biomolecules and dynamically regulate biochemical activity across physiology and pathology.^1^ While studies of cellular condensates have clarified how multivalent interactions among intrinsically disordered proteins and nucleic acids drive assembly, parallel evidence indicates that viral replication cycles exploit related physical chemistry.^2,3^ For instance, retroviral nucleocapsid (NC) proteins compact and dimerize genomes, chaperone folding, and mediate assembly through a combination of electrostatic interactions, aromatic contacts, and sequence-specific RNA motifs.^4,5^ Understanding which sequence-encoded nucleic-acid features tune nucleic acid-peptide-driven coacervation is central both to viral biophysics and to the rational design of programmable condensates.^6–8^

To date, complex coacervation systems are mainly focused on RNA^9–11^ or RNA/peptide phase separation,^12–18^ leaving open how programmable nanoscale structural properties including local base pairing, stacking, and palindromic dimerizers control peptide-nucleic acid LLPS.^19^ Single-stranded DNA (ssDNA) as an anionic building block offers complementary advantages as a model^20–23^ as it is sequence programmable,^24,25^ thermally tractable,^26^ and amenable to systematic variation of local order and linker motifs.^12,26–29^ Moreover, short palindromic elements in viral genomes can nucleate genome dimerization, thereby enhancing multivalency. Thus, a reductionist DNA/peptide system can directly probe mechanisms that parallel viral genome organization while enabling precise control of molecular parameters.

Despite growing interest in biomolecular condensates, a general framework linking the programmable nucleic acid structure at the single-molecule level to emergent DNA–peptide condensate formation and material properties is still lacking. In particular, it remains unclear whether rational nucleic acid design principles can be used to predict and control LLPS behavior across length scales or how minimal cationic peptides reshape these relationships. Here, we bridge DNA intramolecular sequence design principles to peptide-based biomolecular condensates. Addressing this gap is essential for both understanding the driving forces underlying viral-induced membraneless organelles and establishing nucleic acids as modular, designable components for synthetic condensates.

In this work, we sought to study how varying levels of DNA nanoscale folding/unfolding and palindromic linkers govern microscale LLPS propensity and condensate material properties and whether a minimalistic HIV-NC-derived cationic peptide can modulate DNA packing and thereby DNA/peptide interactions. To address these questions, we combined a minimalistic HIV-NC-derived cationic peptide with a designed ssDNA library spanning sequences with varying levels of folding and palindromic architectures, integrating spectroscopic, structural, computational, and phase-behavior analyses to connect nanoscale DNA organization with emergent condensate properties. We found that increasing DNA structural order suppresses LLPS propensity, whereas disordered architectures combined with palindromic linkers promote condensate formation and increased viscosity. These effects depend on both DNA architecture and peptide chemistry, revealing complementary roles of structural flexibility and specific interactions in driving the assembly. Together, these results identify two simple, programmable design parameters, local folding/unfolding and palindromic dimerizing elements, that map nanoscale nucleic acid organization onto micrometer-scale condensate formation and material properties. The findings shed light on potential mechanisms by which NC proteins regulate genome compactification and packaging and provide practical design rules for engineering DNA/protein condensates as tunable microenvironments.

## Results and Discussion

### Design of HIV-1 NC-Derived Peptides and DNAs with Varying Level of Order/Disorder

Inspired by the chaperone activity of the HIV-1 nucleocapsid (NC) protein, which promotes viral genome folding and packaging during the assembly process by binding to the viral RNA and regulating its folding state,^2,30^ we sought to investigate whether a minimalistic peptide derived from HIV NC protein, can alter the folding state, or the level of order/disorder of nucleic acid chains and, in turn, regulate their complex coacervation and formation of biomolecular condensates. As a model system, we designed a cationic 13-mer minimalistic peptide derived from the N-terminal region of HIV NC, a region which is involved in phase separation.^2,31,32^ The peptide, termed HIV nucleocapsid peptide (HNP), comprises residues 2–14 of HIV NC,^5^ excluding the initial methionine. We focused on single stranded DNA (ssDNA) oligos as a model system due to their higher stability and compatibility compared to RNA oligos. We reasoned that multivalent interactions between the NC-derived peptides and ssDNAs of varying structural order could induce conformational changes that regulate the formation and properties of biomolecular condensates.^33,34^

To create ssDNAs with defined levels of order/disorder, we chose tRNA as a structural model. We reverse-transcribed a human tRNA sequence while conserving its GC content, generating four sequences with identical base composition but distinct arrangements. These were termed DNA0, DNA50, DNA75, and DNA100, corresponding to the level (by%) of predicted structural order (Figure 1a, b, Table S1). DNA100 was designed as the fully ordered construct containing one stem and three hairpins. Each stem was generated from a random sequence paired with its complementary strand, while each loop was randomly assigned. Secondary structure prediction using UNAfold^35^ confirmed that DNA100 folds into a tRNA-like cloverleaf structure (Figure S1). Upon addition of 40 mM of Mg^2+^, the predicted free energy (ΔG) of DNA100 became more negative by 10.48 kcal mol^–1^ (Table S2), and the melting temperature (*T*_m_) increased by 18.9 °C, indicating Mg^2+^-dependent stabilization.

**Figure 1:**
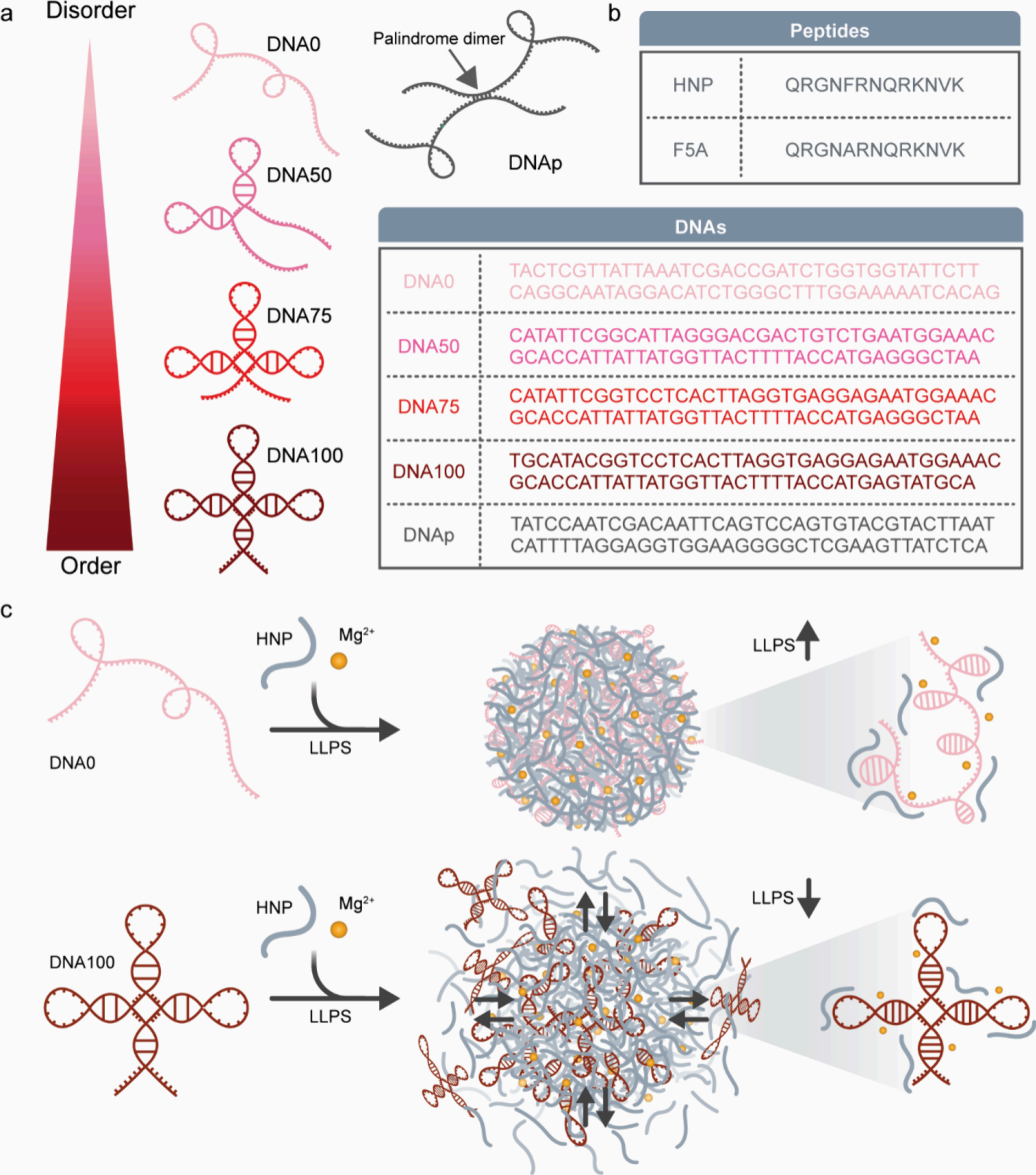
Programmable DNA order/disorder controls DNA/peptide phase behavior. (a) Schematic representation of the disorder-to-order scale bar and the predicted secondary structures or random structure of the designed DNA oligos: DNA0, DNA50, DNA75, and DNA100. Palindrome-containing DNAp (gray) forming a dimer is presented on the right. (b) Sequences of HIV NC-inspired peptide (HNP) and the variant F5A and designed DNAs, with the colored code of the latter corresponding to the predicted structures in (a). (c) Schematic illustration of the designed system showing the phase separation of disordered DNA (DNA0, light pink) and ordered DNA (DNA100, dark red) with the HNP and in the presence of Mg^2+^ ions. DNA100 forms weaker intermolecular interactions with the peptide, while the flexible DNA0 forms stronger intermolecular interactions with HNP.

For the least ordered sequence, DNA0, we selected a random ssDNA from a pool of 100 shuffled sequences according to two criteria: (1) the highest ΔG value, reflecting minimal predicted stability, and (2) the absence of palindromic segments of six or more nucleotides to prevent intermolecular pairing (Supporting Information). DNA0 lacks such palindromes and is predicted to form only three short hairpins with Mg^2+^ (ΔG = −3.82 kcal mol^–1^, Table S1, Figure S1), showing little change in ΔG or *T*_m_ with Mg^2+^, consistent with an unstructured molecule (Table S2). Among the random pool, DNA0 had the second-highest ΔG. The sequence with the highest ΔG (1.98 kcal mol^–1^, Figure S1, Table S2) contained an 8-nucleotide palindrome (GTACGTAC) predicted to form dimers. This sequence was included as a separate construct, termed DNA-palindrome (DNAp), to test the effect of order/disorder on LLPS (Figure 1a, b). The partially ordered constructs were designed based on DNA100 and DNA0 to systematically vary structural order: DNA50 includes hairpins 2 and 3 from DNA100, with the remainder identical to DNA0, whereas DNA75 retains all three hairpins from DNA100 but replaces the stem region with that of DNA0 (Figure S1).

Next, we examined whether these sequence-encoded architectures differ in their ability to undergo LLPS with the HNP. We find that DNA folding propensity is inversely correlated with phase separation: compact, highly folded constructs exhibit reduced LLPS, whereas disordered architectures and palindromic sequences display strongly enhanced phase separation (see in detail, in the LLPS section).This global relationship motivated the detailed structural, spectroscopic, computational, and phase behavior analyses presented below.

### Varying Levels of DNA Compactization by Design

We analyzed the level of compactness of the designed ssDNAs using circular dichroism (CD). For this, we used low micromolar DNA concentrations (5 μM) in an attempt to specifically focus on intramolecular DNA folding and avoid intermolecular interactions. Samples were made in Tris-HCl buffer at pH 8. All five DNAs showed a characteristic structural DNA ellipticity maximum at λ = 274 nm (Figure S2). Increasing the temperature to 90 °C decreased the intensity of the 274 nm maxima and increased the intensity of the λ=248 nm minima (Figure S2a–e). Since the 270–280 nm CD maxima are attributed to π–π stacking and H-bonding in DNA, it is likely that larger deltas of these peaks upon heating is a result of a less-stable secondary structure. Addition of MgCl_2_ at a DNA:salt molar ratio of 1:300 mildly increased the Δ of this trend (Figure S2f–j).

We performed a CD renaturation test where the DNAs were rapidly heated to 90 °C and then cooled down to 5 °C at 1 °C/min (Figure 2a, b). Upon completion of the cooling process, the ellipticity intensity of DNA0 and DNAp increased only by 34.6% and 21.6%, respectively, compared to the intensity during heating, suggesting that no drastic structural changes occur upon heating/cooling. Addition of MgCl_2_ increased this delta to 42.5% and 32.2%, for DNA0 and DNAp, respectively (Figure 2a, b). In contrast, a 65.2%, 60.1%, and 49.0% increase in ellipticity intensity upon cooling was observed for the more ordered DNA oligos, DNA50, DNA75, and DNA100, respectively, indicating that these DNA oligos underwent significant structural changes upon heating/cooling. These deltas increased even further to 110.2%, 103.6%, and 89.2% for DNA50, DNA75, and DNA100, respectively, with MgCl_2_ (Figure 2a, b). The calculated melting temperature (*T*_m_) derived from the renaturation plots showed a clear trend depending on the predicted level of order, with *T*_m_ = 26.4 ± 3.8 °C, 31.9 ± 2.7 °C, and 32.2 ± 3.5 °C for DNA50, DNA75, and DNA100, respectively (Figure 2c). The *T*_m_ values increased to 50. 0 ± 1.4 °C, 44.9 ± 3.3 °C, and 49.5 ± 1.5 °C, for DNA50, DNA75, and DNA100, respectively (Figure 2d) indicating enhanced stability of the ordered and partially ordered DNAs structure upon Mg^2+^ ion coordination.^36^ The minor change in ellipticity as a function of temperature of DNA0 and DNAp suggests their secondary structure is less stable, as suggested by the predicted ΔG. *T*_m_ values were not obtained for these DNAs as their data did not fit the classical renaturation/denaturation model. The significant differences in the CD ellipticity trends between the DNAs that were predicted to be structured and those that were predicted to be unstructured confirm that they adopt varying levels of structural order/disorder. Although the differences in ellipticity and *T*_m_ values among DNA50, DNA75, and DNA100 are relatively small, the secondary structure of DNA50 may adopt a more stable topology in the presence of Mg^2+^. The absence of a significant difference between DNA0 and DNAp in the CD renaturation assay indicates that these conditions do not provide evidence for a dimer-related structure of DNAp.

**Figure 2:**
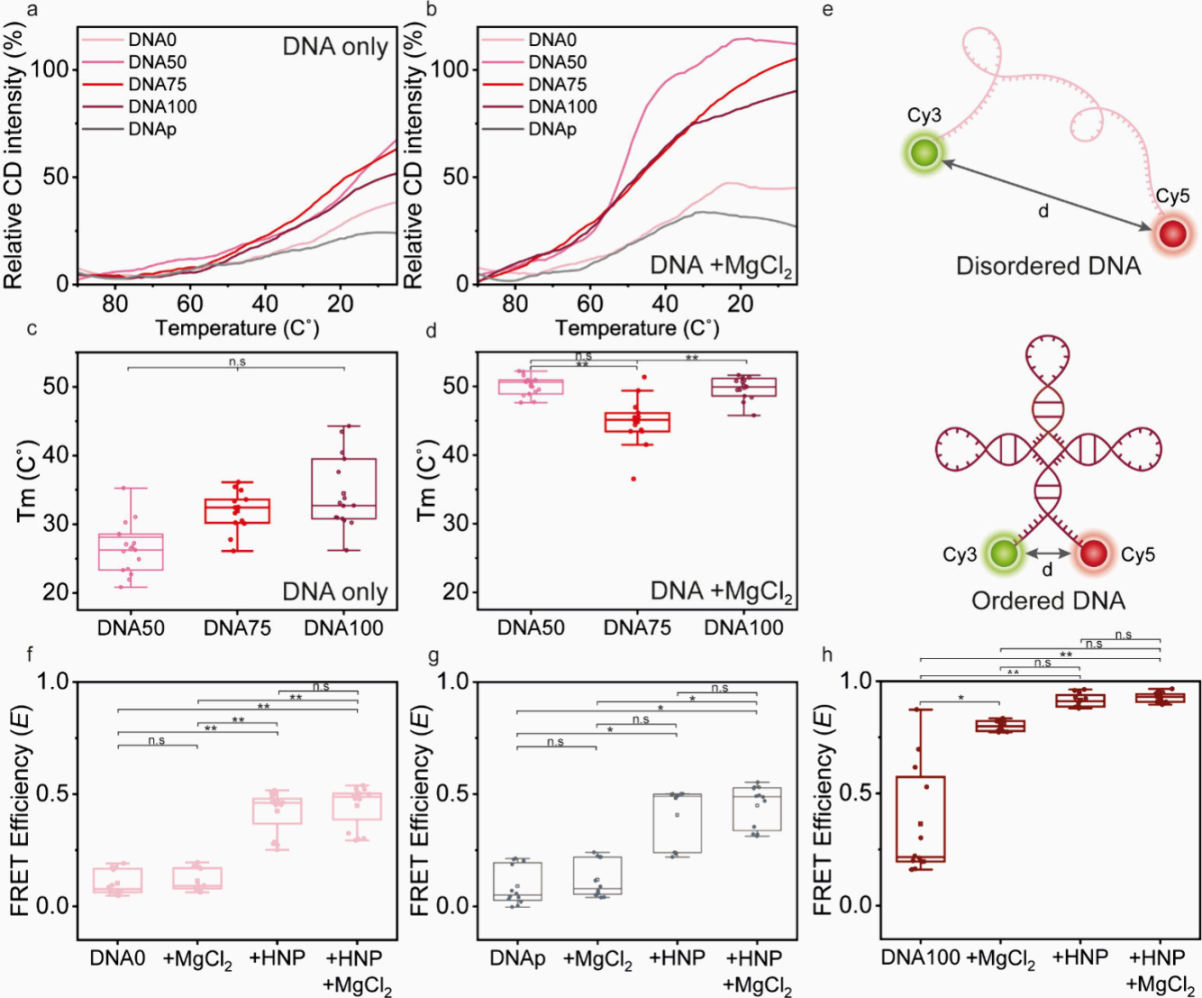
Structural order/disorder is encoded by the DNA sequence and can be modulated through interactions with HNP peptide. (a–d) CD renaturation plots (a, b) and calculated melting temperatures (*T*_m_) (c, d) of the various DNAs in the absence (a, c) or presence (b, d) of MgCl_2_ (1.5 mM). Analysis was performed by monitoring the ellipticity intensity at λ = 274 nm across decreasing temperatures from 75 to 5 °C. Values represent average (a, b) or boxplots (c, d) from three independent analyses; boxplots are based on *n* = 15. (e) Schematic illustration of DNA0 and DNA100 conjugated to the Cy3-Cy5 FRET pair at the 5′ and 3′ ends, respectively, showing the FRET distance (d). (f–h) Cy3-Cy5 FRET efficiency (E) analysis of DNA0 (f), DNAp (g), and DNA100 (h) either alone or in the presence of MgCl_2_ (300-fold excess), HNP (1:1 charge ratio) or both, at 25 °C. Values are presented as box plots, *n* = 12 from three independent analyses.

We further studied the level of DNA packing by fluorescence resonance energy transfer (FRET) analysis, focusing on the most ordered DNA, DNA100, and the least ordered DNAs, DNA0 and DNAp. To analyze the folding and compactization state of the DNAs, we monitored the fluorescence of the Cy3/Cy5 pair, which was conjugated to the 5′ and 3′ ends of the DNAs, respectively (Figure 2e). DNA folding to a more compact structure should lead to a closer proximity of the 5′ and 3′ ends, resulting in an increase in FRET efficiency (E) from Cy3 to Cy5 and a decrease in the FRET distance (d), which were calculated based on the fluorescence of Cy3-labeled DNA and Cy3-Cy5 FRET pair-labeled DNA (Figure S3). To reduce the likelihood of intermolecular contributions to FRET, we used a nanomolar concentration (50 nM) of each DNA in Tris-HCl buffer at pH 8. The calculated E of DNA0 (E = 0.10 ± 0.05) and DNAp (E = 0.09 ± 0.08) were very similar, corresponding to d of 7.5 ± 0.68 nm and 8.32 ± 2.23 nm (Figure 2f, g, Table S3). In contrast, DNA100 showed a higher E of 0.36 ± 0.24, corresponding to a shorter d of 5.76 ± 0.98 nm (Figure 2h), confirming that DNA100 adopts a more compact and ordered structure. The near-zero E of DNA0 and DNAp suggests greater conformational variability and structural flexibility. Thus, addition of 300-fold molar excess MgCl_2_ barely changed the E of DNA0 and DNAp (ΔE = 0.01, 0.03, respectively, Figure 2f, g), whereas it doubled the E of DNA100 (ΔE = 0.44, Figure 2h, Table S3). These results indicate that DNA100 undergoes structural compactization by interaction with MgCl_2_. The findings support the DNA sequence design, with charge screening by Mg^2+^ ions facilitating compactization of the already ordered DNA100, owing to its inherent H-bonding compatibility. In contrast, DNA0 and DNAp remain mostly unfolded, as predicted, even upon charge screening by Mg^2+^ ions due to lack of H-bonding compatibility.^15^

### HNP Peptide Induces DNA Compactization

We studied the effect of the HNP peptide on DNA structure using FRET. For this, we added the peptide at a 1:1 DNA:peptide charge ratio. At these nanomolar concentrations (peptide concentration is 740 nM), DNA dimerization, peptide–peptide interactions, or LLPS are not likely to occur. HNP increased the E of DNA0 and DNAp by 4.0-fold (ΔE = 0.3) and 4.5-fold (ΔE = 0.32), respectively (Figure 2f, g). Notably, a nearly maximal E (0.92 ± 0.03, ΔE = 0.55) was observed for DNA100 upon addition of HNP (Figure 2h, Table S3), indicating that the peptide strongly promotes compactization of the DNA chain. Similar values were observed upon applying both HNP and MgCl_2_. These results show a significant reduction in FRET distance in the presence of HNP for all DNAs, especially DNA100, suggesting that the peptide induces a chaperone-like effect on the DNA chains, possibly guiding the folding of both ordered and disordered DNAs toward more compact conformations. This suggests that HNP is forming peptide–DNA assemblies reminiscent of viral ribonucleoprotein structures.^5^

To assess whether the observed peptide-induced DNA compaction persists in a more crowded environment, which mimics the molecular crowding during the viral infection cycle, we performed FRET measurements on the DNA0-HNP system in the absence and presence of a macromolecular crowder (Figure S4a–c). Dextran was added at a final concentration of 20 w/v%, approximating intracellular macromolecular crowding. Crowding alone resulted in only a modest increase in FRET efficiency (ΔE = 0.08), whereas the combined presence of dextran and HNP led to a significant increase in FRET efficiency (ΔE = 0.41), suggesting an additive contribution of crowding and peptide-mediated interactions (Figure S4c).

Furthermore, we aimed to learn whether HNP-induced compaction arises solely from nonspecific electrostatic interactions or involves composition-dependent contributions. We performed additional comparative FRET measurements. Specifically, we compared DNA0 compaction induced by HNP with that induced by two control peptides at matched 1:1 charge ratios: (i) F5A, a phenylalanine-to-alanine variant of HNP, and (ii) VFP-1, a viral-factory-inspired 14-mer oligo-cationic peptide (LGKSG­RLPGK­SGRV, net charge +4), previously reported by us.^33^ HNP induced a higher FRET efficiency (E = 0.46 ± 0.01) than F5A (E = 0.41 ± 0.01) and significantly higher than VFP-1 (E = 0.30 ± 0.03). While all three peptides can induce electrostatically driven DNA compaction, these results demonstrate that the presence of an aromatic residue in HNP enhances compaction beyond what can be achieved by charge alone. This finding indicates that DNA compaction in this system is not governed exclusively by nonspecific electrostatic attraction but instead involves composition-dependent interactions, which are likely mediated by π-stacking contributions and hydrophobic interactions. Consistent with this interpretation, previous studies have shown that the N-terminal region of the HIV-1 NC protein plays a key role in viral RNA binding and organization.^37^ Specifically, the hydrophobic environment induced by the side chains of Phe at position 6 and Val at position 13 contributes to RNA binding.^38^ Taken together, these results support a model in which HNP promotes nucleic acid compaction through multivalent, sequence-encoded interactions rather than classical chaperone-like folding into a specific ordered structure. More broadly, they suggest that similar compaction mechanisms may operate in intracellular environments during viral infection and that the minimal DNA/peptide system employed here provides a useful platform for probing the dynamics of viral nucleic acid polymers.

We analyzed DNA folding in the presence of HNP using small-angle X-ray scattering (SAXS). DNA samples were heated in a flow cell from 25 to 80 °C (above the DNA melting temperature), cooled back to 25 °C, and measured again. After cooling, the Kratky plot of DNA100 shows signatures of folding into a rigid-body structure (Figure S5a). Guinier analysis reveals that the radius of gyration (Rg) of DNA100 increases from 1.8 ± 0.1 to 2.2 ± 0.5 nm upon HNP addition, indicating the formation of larger DNA complexes, upon DNA-HNP complexation (Figure S5a, Table S4). In contrast, the Rg of DNA0 slightly decreases from 2.1 ± 0.1 to 1.95 ± 0.04 nm upon HNP addition, suggesting HNP-assisted folding (Figure S5b). No significant change is observed for DNAp, with Rg values of 2.34 ± 0.05 nm and 2.37 ± 0.08 nm in the absence and presence of HNP, respectively (Figure S5c). Unlike FRET, SAXS reports ensemble-averaged structural properties rather than specific intramolecular distances. Nonetheless, consistent with the FRET data, pair distance distribution functions (PDDFs) indicate that HNP complexation leads to more elongated structures and increased Porod volumes (Figure S5d–f). For DNA100, the maximum particle dimension (*R*_*max*_) shifts from 5.55 to 8.56 nm upon HNP binding, accompanied by a broader PDDF (Table S4). For DNA0, *R*_*max*_ increases from 6.8 to 7.4 nm despite an overall compaction of the ensemble. For DNAp, *R*_*max*_ increases modestly from 7.6 to 8.0 nm, along with an increase in Porod volume from 24.3 to 36.9 nm^3^. Together, these observations indicate distinct modes of DNA–peptide complexation: HNP binding induces ensemble compaction in flexible DNA0, reconciling its reduced Rg with a smaller *R*_*max*_, while decorating and expanding the outer dimensions of the more rigid DNA100 scaffold. In contrast, HNP association with DNAp likely occurs within internal voids, resulting in minimal changes to overall dimensions as captured by SAXS.

### Computational Analysis of DNA Structure, Stability, and Compactization

We subsequently studied the compactization and structural stability of DNAs and peptide–DNA interactions by coarse-grained molecular dynamics (CGMD) simulations (Tables S5–7) to provide molecular-level context for the experimentally observed phase behavior and condensate properties (Figure 3). Representative DNA structures depicted their most probable conformations in DNA-only systems (Figure 3a and Figure S5). We further examined the structural ensemble of the DNAs and used normalized Rg and solvent-accessible surface area (SASA) as indicators (Figure 3b–f). Rg focuses on the overall DNA size and flexibility, while SASA focuses on DNA-solvent interactions and DNA hydrophilicity. DNA0 and DNA75 without Mg^2+^ ions or peptides showed the most discrete SASA-Rg distribution, suggesting an absence of a specific conformational state (Figure 3b, d). In contrast, DNA50, DNA100, and DNAp-only systems showed more compact distributions, which indicates the existence of stable secondary structures (stems and hairpins) with less freedom (Figure 3c, f).

**Figure 3:**
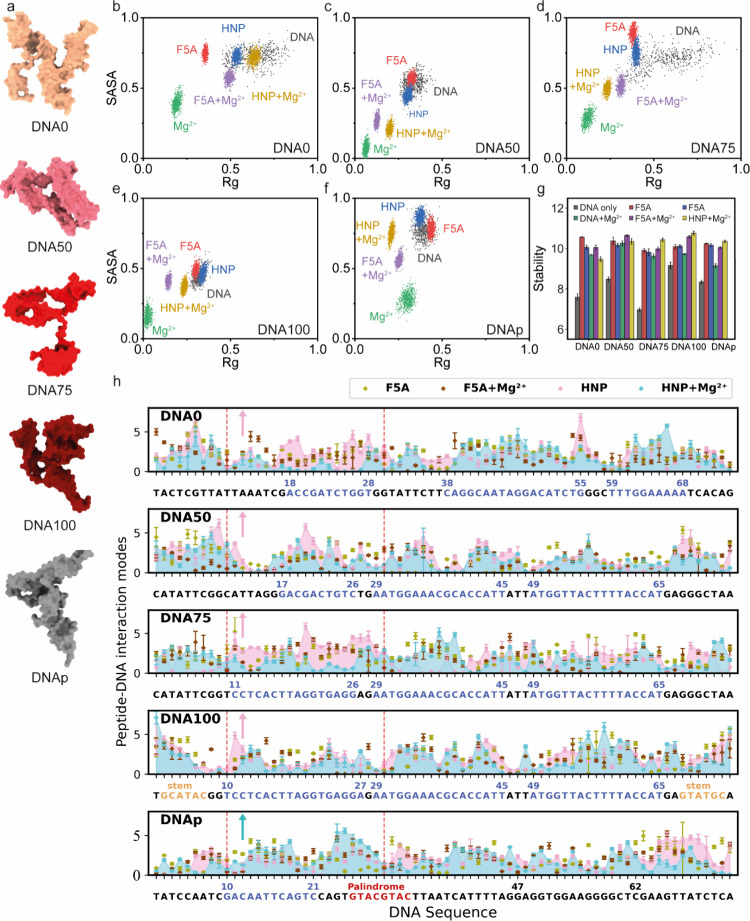
Compactization, structural stability of DNAs, and DNA–peptide interaction modes analyzed by coarse-grained molecular dynamics simulations. (a) Representative structural snapshots of DNAs. (b–f) Distributions of DNAs’ structural features. Smaller SASA and Rg indicated stronger folding and compactization. (g) Structural stability of DNAs, a 3-fold random subsampling was used to estimate stability and standard deviation. (h) DNA–peptide interaction modes. The *x*-axis showed DNA sequences annotated with secondary structures, including stems, hairpins (blue) and palindromic linker, and the *y*-axis represented the bead number of coarse-grained peptide aggregating to DNA. Pink (without Mg^2+^) and cyan (with Mg^2+^) filling represented HNP-DNA interactions.

With the addition of Mg^2+^ ions or peptides, the compactness of DNAs, characterized by Rg and SASA, showed a significant difference compared to the DNA-only system (Figure 3b–f). Specifically, DNAs exhibited minimal Rg and SASA with the addition of Mg^2+^ ions, showing the most compact structure. Conversely, in the peptide-containing systems, Rg and SASA remained comparable to those in the DNA-only system, suggesting no significant increase in structural compactness. Similarly, the internal contacts of DNA structures further illustrate the influence of peptide/Mg^2+^ on its conformation (Figure S6), for example, in the DNA + Mg^2+^ system, the average contact probability of DNA residues increased markedly, whereas only a modest increase was observed in the presence of peptides (Figure S7). This observation indicates that Mg^2+^ primarily facilitated DNA folding, whereas peptides, probably due to steric hindrance, mainly promote DNA cross-linking. A more compact structure seems to correlate with greater stability, a finding supported by our structural stability calculations (Figure 3g). The increased structural stability was induced by electrostatic interactions with Mg^2+^ ions (Figure S8) or noncovalent binding with peptides (Figure 3h). Both mechanisms contributed to an enhanced structural stability, indicating the formation of stable complexes that favor phase separation.

#### Distinct Mechanisms of FRET Enhancement by Mg^2+^ and Peptides

Higher FRET efficiency reflects a sufficiently short end-to-end distance between fluorophores and effective dipole orientation.^39^ In CGMD simulations, sustained correct dipole orientation correlates with stable conformational ensembles. After incorporating structural stability into the FRET calculations, we found that systems containing peptides, with or without Mg^2+^, exhibited higher FRET efficiencies than systems containing Mg^2+^ alone, which, in turn, exceeded DNA-only systems. Moreover, DNA100-containing systems consistently showed higher FRET efficiencies than DNAp-containing systems (Figure S9, Table S8). In HNP-containing systems, DNA100 exhibited normalized (Rg, SASA) values of approximately (0.3, 0.5), whereas DNAp showed values of approximately (0.3, 0.9). In the presence of both HNP and Mg^2+^, these values shifted to approximately (0.25, 0.35) for DNA100 and (0.25, 0.75) for DNAp. Despite similar Rg values, the substantially lower SASA for DNA100 (Figure 3e, f) indicates a more compact structure relative to DNAp, resulting in reduced FRET distances and increased FRET efficiency. The modest additional decrease in both Rg and SASA upon Mg^2+^ addition to HNP systems accounts for the slightly higher FRET efficiency compared with HNP alone. These trends are consistent with the experimentally measured FRET results (Figure 2g, h).

Notably, although Mg^2+^ alone induces more compact DNA folding and, consequently, shorter FRET distances, peptide-containing systems still exhibit higher FRET efficiencies. This indicates that peptides more effectively promote favorable dipole orientations and stabilize DNA conformations conducive to efficient energy transfer beyond compaction alone.

#### Sequence-Specific Peptide Binding and Concentration Dependence

Peptide–DNA interaction profiles provide insight into how local sequence features contribute to molecular aggregation during phase separation or condensation (Figure 3h). Across all DNA constructs, peptide binding is distributed over multiple regions rather than being confined to a single site. Notably, DNAp exhibited enhanced HNP association at nucleotides 10–30 in the presence of Mg^2+^ (marked pink region, Figure 3h), which includes the palindromic sequence GTACGTAC. In contrast, DNA100 also shows pronounced peptide binding in ordered regions, including nucleotides 49–65, but these interactions occur within a structurally rigid scaffold. The preferential enhancement at palindromic sites within the fully disordered DNAp architecture suggests a distinct interaction mode compared to that of ordered DNA100, consistent with increased interaction heterogeneity rather than stronger binding per se. Additionally, we observe competitive binding between peptides and Mg^2+^ across multiple regions, indicating that peptide–DNA association, and by extension LLPS propensity, is sensitive to both peptide and Mg^2+^ concentrations. In systems containing both peptides and Mg^2+^, increased populations of free peptides and ions are observed (Figure S8), despite simulations reaching equilibrium on the microsecond time scale (Figure S10). Mg^2+^ conversely reduces peptide–DNA aggregation in several regions (Figure 3h), supporting a competitive equilibrium that likely underlies the concentration dependence of LLPS.

#### DNAp Dimerization and Structural Characterization

Coarse-grained simulations further suggest that DNAp can form Mg^2+^-stabilized dimers with extended, solvent-exposed conformations that increase peptide-accessible surface area, potentially enhancing multivalency. Detailed structural characterization of those dimers is provided in the Supporting Information (see Supplementary Computational Discussion and Figures S11–S17).

### Phase Behavior of DNA/Peptide Condensates

While DNA conformations within condensates are not assumed to be identical with those observed under dilute conditions, sequence-encoded structural tendencies are expected to bias interaction patterns and multivalency during condensate formation. To comprehensively characterize LLPS, we combined phase diagrams, turbidity assays, microscopy, FRAP, and fusion dynamics, enabling an assessment of phase boundaries, robustness, reversibility, and material properties.

First, we sought to elucidate how our DNA designs affect the capacity of the DNAs to complex and phase-separate with HNP. For this, we generated phase diagrams based on microscopy analysis as a function of DNA/peptide and MgCl_2_ concentrations. To exclude contributions of charge repulsion caused by excess charge, we kept a 1:1 charge stoichiometry of DNA:peptide across all samples. Samples were formed in a Tris-HCl buffer at pH 8.

Overall, a positive correlation was observed between the expected level of order and the saturation concentration (C_sat_) with increasing level of order resulting in an increase in the C_sat_ and a decrease in the LLPS propensity, as expressed by the two-phase area (Figure 4a–d). Specifically, the most disordered DNA, DNA0, undergoes LLPS with HNP with a C_sat_ of 25 μM, where the two-phase region encompasses a wide range of MgCl_2_ concentrations between 0 and 15 mM. DNA50 and DNA75 showed a 2-fold higher C_sat_ of 50 μM and a similar two-phase area, spanning 0–10 mM MgCl_2_ for DNA50 and 2.5–12.5 mM MgCl_2_ for DNA75 (Figure 4b, c). The highest C_sat_ of 75 μM was observed for DNA100 (Figure 4d), which showed a much narrower two-phase region spanning 2.5–7.5 mM MgCl_2_. Surprisingly, the palindrome-containing DNA, DNAp, showed the highest LLPS propensity, with a C_sat_ of 12.5 μM and a significantly wider two-phase region ranging from 1 to 50 mM MgCl_2_ (Figure 4e). The gradual increase in C_sat_ as the predicted level of order increases indicates that the compactization of the DNA plays an important role in LLPS. In addition, the difference between the two disordered DNAs, DNA0 and DNAp, suggests that the dimerization of the disordered DNA facilitates interactions with HNP and, in turn, LLPS.

**Figure 4:**
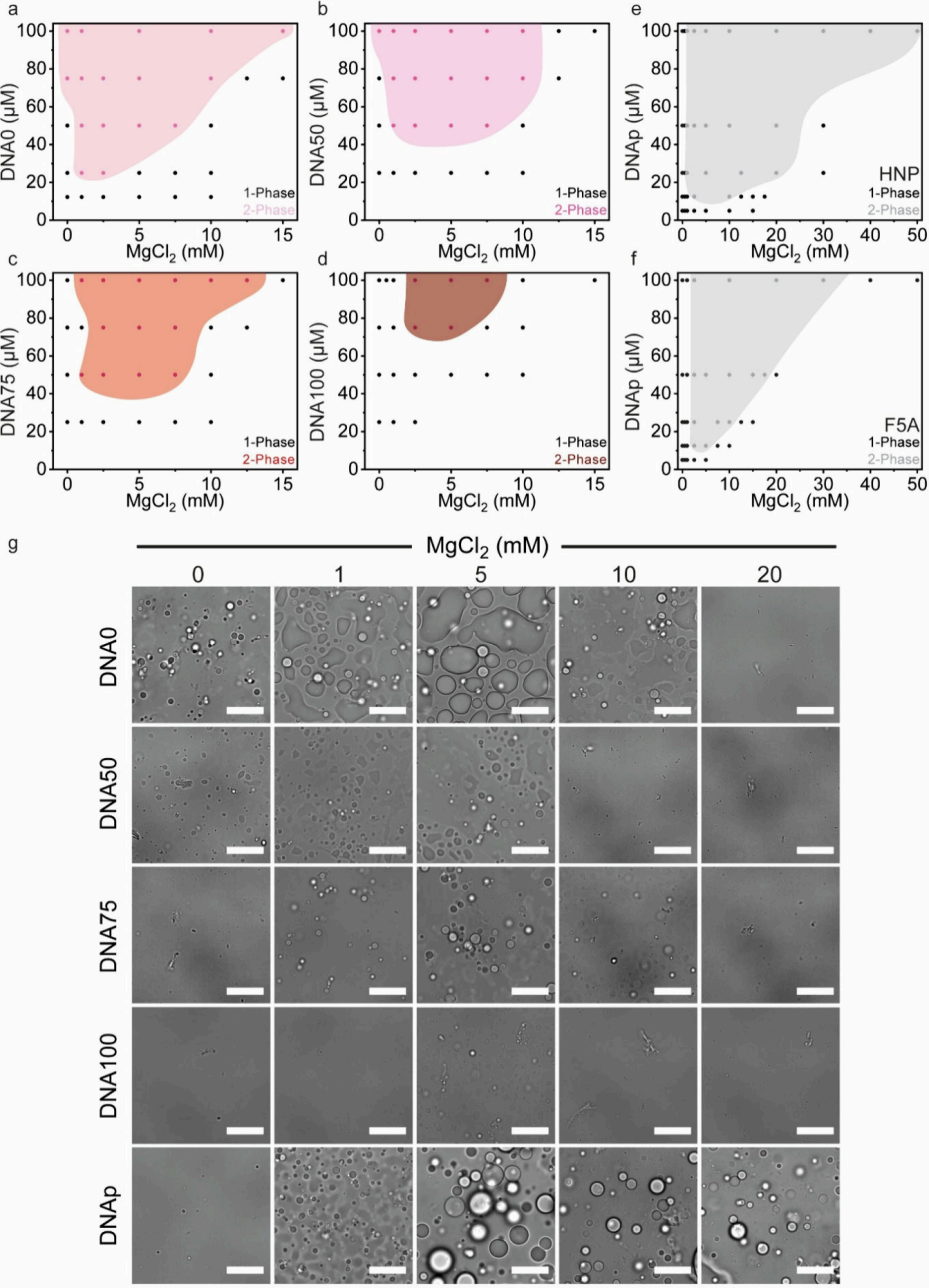
The phase separation propensity of DNA/HNP is directly encoded by the structural order/disorder of the DNA. (a–f) Phase diagrams of DNA0 (a), DNA50 (b), DNA75 (c), DNA100 (d), and DNAp (e, f) as a factor of MgCl_2_ concentration and DNA/peptide concentration (1:1 charge ratio). (g) Brightfield micrographs of DNA/HNP solutions at varying MgCl_2_ concentrations. Images show 100 μM DNA at DNA/peptide 1:1 charge ratio. Scale bars = 10 μm.

To assess the contribution of π–π and cation−π interactions to DNA/peptide complexation, we introduced a phenylalanine-to-alanine substitution at position 5, generating the variant HNP-F5A (Figure 1b). Strikingly, except for DNAp, all DNAs failed to phase separate with F5A across all tested conditions (12.5–100 μM DNA and 0–50 mM MgCl_2_). In contrast, DNAp underwent LLPS with F5A with a similar propensity to that observed for HNP, with a C_sat_ of 12.5 μM and a two-phase region spanning 2.5–30 mM MgCl_2_ (Figure 4f). Thus, omitting one phenylalanine residue from the peptide sequence resulted in a complete inhibition of LLPS for all the DNAs, suggesting that π–π and cation-π interactions^16^ facilitated by phenylalanine side chains are critical for DNA/peptide LLPS and that the latter does not merely rely on electrostatic complexation, as typically attributed for polycations and polyanions. The ability of DNAp to interact and undergo LLPS with F5A might indicate that the disordered and spread conformation of DNAp, facilitated by palindromic linker and dimer formation, significantly contributes to the multivalency which is critical for LLPS.

To examine the robustness of DNA/peptide interactions underlying LLPS, DNA/HNP condensates were next subjected to increasing urea concentrations (0–500 mM, Figure S18). To ensure observable condensates across all DNA variants, experiments were performed at 75 μM DNA, 740 μM HNP, and 5 mM MgCl_2_. Microscopy images revealed a progressive decrease in condensate abundance with increasing urea concentration (Figure S18a). Consistently, turbidity measurements showed an overall decrease in LLPS propensity, with the notable exception of DNAp condensates (Figure S18b). Accordingly, the relative turbidity decreased by 45.7% (±7.3), 61.6% (±1.1), 52.8% (±2.1), and 63.2% (±7.8) for DNA0, DNA50, DNA75, and DNA100, respectively, at 0.5 M urea. Notably, the response of DNA50 more closely resembled that of DNA100 than DNA0, further supporting the idea that DNA50 adopts a folded structure. In contrast, DNAp exhibited only a minimal turbidity decrease of 4.2% (±0.6), highlighting its distinct LLPS behavior and enhanced stability.

As a complementary approach, we further examined how macromolecular crowding modulates DNA/peptide LLPS to approximate intracellular conditions relevant to viral infection. The LLPS propensity of DNA0/HNP condensates was analyzed both microscopically and using turbidity measurements across increasing dextran concentrations (Figure S19). Experiments were conducted at a relatively low DNA0 concentration selected from the phase diagram (50 μM DNA0, 740 μM peptide, and 5 mM MgCl_2_; Figure 4a). Increasing dextran concentration led to a pronounced increase in droplet size and density over 1 h (Figure S19a). Consistently, turbidity measurements showed a gradual enhancement of LLPS with dextran concentration, approaching a plateau at 20% (w/v) (Figure S19b). These results show that molecular crowding resembling the intracellular environment strongly amplifies DNA/peptide interactions. We further analyzed how intracellular physiological density and ionic strength affect the viability of DNAp, DNA0, and DNA100 condensates with HNP (Figure S20). For this, we used 20 mM HEPES buffer (pH 7.5) with 150 mM salt and 20% w/v dextran. At 50 μM, none of the DNAs underwent phase separation under this high ionic strength. At 100 μM (with HNP 1.48 mM at a 1:1 charge ratio), the high ionic strength suppressed phase separation of DNA0 and DNA100, but not that of DNAp (Figure S20a, b). These results highlight the higher LLPS propensity of DNAp compared to the other DNAs and the relevance of our findings to broader conditions including cellular environments.

In the absence of HNP, the DNAs alone exhibited micrometer-sized aggregates, which were most prominent in DNA100 (Figure S21, Figure 4g). At the border of the two-phase region, DNA100 showed low abundant droplets adhered to rod-shaped aggregates (Figure S22, Figure 4g). Overall, DNAp–peptide condensates were significantly more abundant than all other DNAs, showing a more turbid liquid mixture (Figure 4g). Interestingly, it seems that DNAp/peptide LLPS requires MgCl_2,_ while DNA0 and DNA50 (100 μM) undergo LLPS with HNP without salt. In addition, DNA75 and DNA100 (100 μM) also showed an immediate and transient turbidity without MgCl_2_ upon mixing with the HNP. This transient phase separation disappeared upon gentle mixing (Figure S23) and when reaching equilibrium transformed into aggregation. The dependency of DNAp/peptide LLPS in Mg^2+^ ions suggest that the folding of the DNA (especially in palindromic linker) into compact conformations, induced by Mg^2+^ interaction with exposed anionic phosphate groups, is critical for facilitating the DNA interaction with peptides.

To assess if the DNA100 aggregates relax at increased temperatures and whether they could transform into condensates, we performed a temperature-dependent microscopy analysis in the presence of 10 mM MgCl_2_ (Figure S24). DNA100/HNP samples were loaded on a glass coverslip on top of a metal temperature-controlled stage and sealed. The samples were heated to 70 °C, incubated for 2 min, and subsequently cooled down to 25 °C (10 °C/min). The DNA100/HNP formed micrometer-sized aggregates that disappear upon heating. No LLPS was observed upon cooling, only micrometer-sized aggregates which settled to the bottom of the well (Figure S24a). In contrast, DNAp condensates, which are formed under these conditions, disappeared gradually upon heating and then re-emerged upon cooling down to 25 °C.

Altogether, these results suggest that the structured and compact nature of DNA100 restricts phase separation and favors aggregate formation over dynamic phase separation, likely due to poor flexibility, structural rigidity, and limited multivalency. The systematic and comparative analysis of the various DNAs shows that both DNA50 and DNA75 possess some degree of structural rigidity, as predicted, but that the level of order of these two DNAs is not substantially different, as detected by the current analyses. In contrast, the structural flexibility of DNA0 facilitates the formation of multivalent interactions with HNPs, resulting in the formation of more abundant condensates.

Our results suggest that DNA0 LLPS is strongly dependent on peptide chemistry and aromatic interactions, while DNAp condensation is dominated by DNA–DNA interactions enabled by palindromic self-association and Mg^2+^-mediated charge screening, with the peptide playing a role in network stabilization. This distinction is supported by the relatively small effect of the phenylalanine-to-alanine substitution in DNAp as well as by the strict requirement for Mg^2+^ to enable DNAp condensation. Moreover, the remarkable stability of DNAp/HNPs in urea suggests a distinctively different network of interactions. Together, these observations show that LLPS in DNAp is driven primarily by a hybrid DNA–DNA scaffold rather than by peptide–DNA interactions alone.

### Material Properties of DNA–Peptide LLPS Condensates

Lastly, we investigated how the DNA levels of order/disorder affect the material properties of the DNA/peptide condensates. For this, we first performed fluorescence recovery after photobleaching (FRAP) analysis for DNA/HNP condensates (with 5 mM MgCl_2_) using 5′-labeled-Cy3-DNA as the fluorescent probe. Substantial differences were observed between DNAp and all other DNAs. Thus, DNA0, DNA50, DNA75, and DNA100 showed a total fluorescent signal recovery of 98.0%, 94.8%, 90.1%, and 95.8%, respectively, while DNAp displayed only 68.9% recovery (Figure 5a, b). The calculated *t*_1/2_ value of DNAp/HNP condensates (15.3 ± 2.4 s) is 5–8-fold larger (Figure 5c) than that of DNA50, DNA50, DNA75, and DNA100 (2.0 ± 0.4 s, 1.9 ± 0.3 s, 3.0 ± 0.6 s, and 2.7 ± 0.3 s, respectively). These results are aligned with the significantly lower C_sat_ and larger two-phase region and LLPS stability observed for DNAp/peptide compared to the other DNAs.

**Figure 5:**
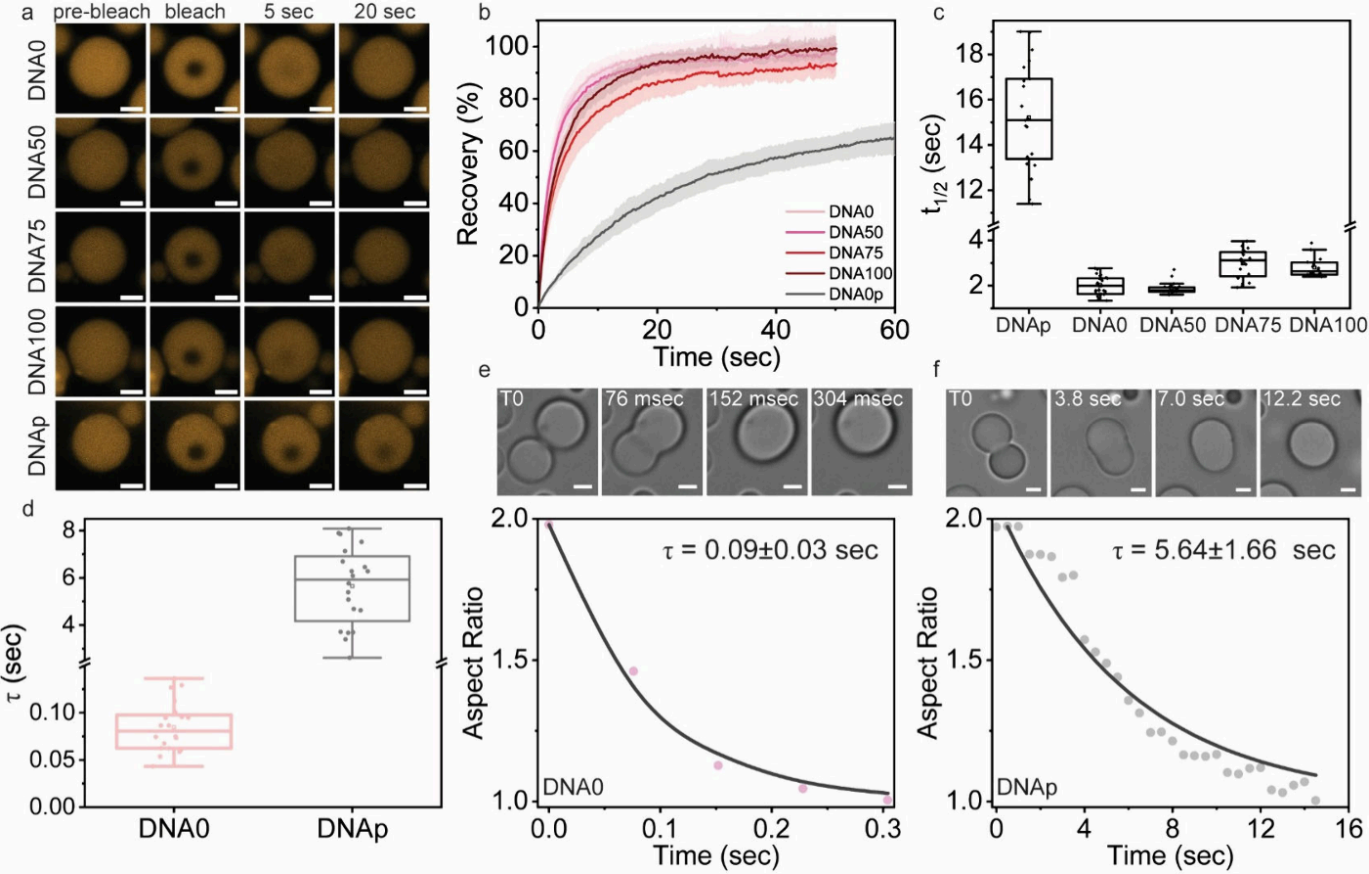
Palindrome-containing DNAp shows remarkably slower diffusivity and dynamics within DNAp/HNP condensates. (a–c) FRAP analysis of the designed DNAs performed by monitoring the recovery after photobleaching of Cy3-labled DNAs. (a) Confocal micrographs of prebleached, immediately bleached, and 30 s postbleaching condensates. Scale bars = 2 μm. (b, c) Recovery plots of DNA/HNP condensates with 5 mM MgCl_2_ (b) and calculated *t*_1/2_ values (c). FRAP analyses show average of three independent analyses, *n* = 27, 19, 20, 17, 20 for DNA0, DNA50, DNA75, DNA100, and DNAp, respectively. (d–f) Condensate fusion assay showing calculated fusion times (τ) of DNA0 and DNAp (d), based on two independent analyses, *n* = 20. (e, f) Representative fusion events with aspect ratio analysis of DNA0 (e) and DNAp (f). Top panels show time-lapse images; bottom panels show aspect ratio decay, with points from experimental measurements and solid lines from exponential fits. Scale bars = 2 μm.

To further investigate this, we performed a condensate fusion assay, where we tested the differences in fusion time (τ, Figure 5d) through changes in fusion aspect ratio of DNA0/HNP (Figure 5e) and DNAp/HNP condensates (Figure 5f), at the same conditions as the FRAP assay. Since almost no fusion events could be detected for DNA100/HNP, we were unable to comparatively analyze this system. Remarkably, the calculated DNAp τ was 62.7 times slower than that of DNA0 (Figure 5d). This significant difference in relaxation time suggests that the dimerization of DNAp induces stronger DNA–DNA and DNA–peptide interactions which slow down the diffusivity and dynamics and increase the viscosity within the dense phase.

## Conclusions

In this study, we demonstrate that the level of DNA structural folding can be rationally predicted, programmed, and leveraged to control DNA/peptide LLPS and condensate material properties. Using a designed ssDNA library spanning a range of folded, partially folded, unfolded, and palindromic architectures, we established that nanoscale DNA folding landscapes directly dictate mesoscale phase behavior. CD and FRET analyses confirm that the designed sequences adopt varying levels of compactness and that these conformational tendencies persist upon interaction with an HIV-NC-inspired peptide, HNP.

A central finding is that DNA folding suppresses LLPS, whereas structural disorder enhances it. Highly folded, compact DNAs favor aggregation over dynamic, liquid-like condensates, reflecting limited flexibility and reduced multivalency. In contrast, disordered and conformationally flexible DNAs more readily engage in multivalent interactions with cationic peptides, promoting robust condensate formation. Notably, introducing a short palindrome sequence linker within the disordered DNA results in a marked increase in LLPS propensity and viscosity as well as an attenuation of diffusivity, suggesting that the combination of sequence disorder and an intermolecular base-pairing region enhances DNA–DNA interactions that promote the formation of an extended DNA-Mg^2+^-peptide network.

Our data further reveal that the role of the HIV NC-derived peptide, HNP, is strongly modulated by DNA architecture and acts as a chaperone-like compaction agent for both ordered and disordered DNAs at the single-molecule level, resembling the chaperone activity of the natural NC protein in promoting the folding and packaging of the viral RNA.^5,40^ This compaction does not universally promote the LLPS. Instead, peptide-driven phase separation emerges only when the DNA flexibility and multivalency are preserved. In the palindrome-containing DNA, LLPS is dominated by DNA–DNA interactions stabilized by Mg^2+^-mediated charge screening, with the peptide serving a secondary, network-stabilizing role. This mechanistic distinction explains the exceptional stability and slow dynamics of these condensates and highlights that peptide chemistry alone is insufficient to predict phase behavior without considering the nucleic acid architecture.

Combined experimental and computational analyses reveal that DNA compaction, driven by Mg^2+^ ions or peptide binding, governs both the phase behavior and the material properties of the resulting condensates. Previous studies have shown that folding of ssDNA into G-quadruplex structures can enhance LLPS.^21,22^ These systems involved LLPS with a large protein (histone H1) that provided structural flexibility through its massive IDRs, while the highly ordered G-quadruplex allowed surface interactions with the protein. In our work, the structural rigidity of the tRNA-like ssDNA limited both flexibility and surface accessibility, whereas a largely disordered DNA enabled both through its palindromic linker and intrinsic structural flexibility. Importantly, recent work demonstrated that DNA palindromic linkers can markedly increase LLPS propensity,^12^ and our findings extend this concept to DNA/peptide systems, showing that such sequences profoundly influence the condensate formation. The internal organization of DNA within the condensed phase cannot be directly resolved from the current data and remains an important subject for future investigations.

Together, these findings indicate that small, nanoscale differences in the level of folding and palindromic dimerization as programmable design parameters of DNA oligomers can translate into large, micrometer-scale changes in condensate formation and material properties. By linking sequence-encoded DNA architecture to phase separation propensity, dynamics, and material state, this work reframes nucleic acid sequence design as a tool to create designable components of biomolecular condensates. These insights provide a mechanistic framework for understanding viral genome organization and offer general design principles for engineering synthetic DNA/peptide condensates with tunable physicochemical properties.

## Materials and Methods

All samples were made in 10 mM Tris-HCl pH 8, using Trizma base (Sigma-Aldrich). Peptides were customed synthesized by Genscript. DNAs were synthesized by Integrated DNA Technologies (IDT). Pluronic F-127/Sigmacote was purchased from Sigma-Aldrich. Dextran from *Leuconostoc mesenteroides* (Sigma) averaged Mw = 9–11 kd. Urea was purchased from Sigma.

### DNA Secondary Structure Predictions

DNA secondary structures, ΔG, and *T*_m_ were predicted using UNAfold^35^ software. All predictions were calculated with 10 mM Na^+^ corresponding to 10 mM Tris +10 mM Mg^2+^ and 25 °C. All shuffled DNA sequences were shuffled using the sequence manipulation suite.^35^

### CD Analysis

CD samples were made with 5 μM DNA, in Tris-HCl buffer pH 8. All CD spectra were measured in a 1 mm quartz sealed cuvette, using a Chirascan spectrometer. Spectra were measured at 90/25/5 °C and incubated for 5 min at each temperature. The background of the Tris-HCl buffer was subtracted from all samples. Raw spectra data were smoothed using a Savitzky–Golay filter (OriginLab software) and averaged from three independent measurements (n = 15).

### CD Renaturation and *T*_m_

Samples were rapidly heated to 90 °C, incubated for 5 min, cooled down to 5 °C, and measured at 1 °C/min. Raw renaturation data were smoothed using a Savitzky–Golay filter and calculated as relative change (%) of 274 nm ellipticity to the 90 °C measurement. *T*_m_ was calculated from a Boltzmann sigmoidal fitted renaturation plot. All data were calculated from three independent measurements (n = 15).

### FRET Measurements

All FRET samples were made in Tris-HCl buffer pH 8, with 50 nM DNA. Samples with peptides were a 1:1 charge ratio with the DNA, samples with MgCl_2_ contained 15 μM. Here, 40 μL of the sample was loaded to each well (four wells for each sample in each of three independent analyses, n = 12) of a 384-well plate, and emission spectra were measured from 566 to 700 nm (λ_ex_ = 540 nm) using a BioTek H1 synergy plate reader. The Tris-HCl background was subtracted from all samples. Here, 5′-Cy3-DNA was measured separately to FRET-DNA.

### FRET Distance Analysis

FRET efficiency (E) was calculated from the FRET sample and donor-only sample emission:

$$E=1-\frac{F_{DA}}{F_{D}}$$

where *F*_*DA*_ and *F*_*D*_ are donor–acceptor and donor-only fluorescence intensities (λ_em_ = 568), respectively. FRET distance (d) was calculated from *E*:

$$d=R_{0}\times {\left(\frac{1-E}{E}\right)}^{1/6}$$

where *R*_*0*_ = 5.1 nm is the Förster distance used from fluorescent protein database.^41^

### Phase Diagrams

All phase diagrams were conducted from brightfield (BF) microscopy using an Olympus IX83 microscope and collected from CellSens Dimension software. Images were taken at 25 °C unless mentioned otherwise. All samples were made in Tris-HCl buffer pH 8, initially making peptide and DNA separate stock solutions and adjusting pH. DNA was added to a Tris + MgCl_2_ solution, and peptide was added last, loaded to a 96-well plate and immediately observed at the microscope. All phase diagram images presented in this article are from 10 min after adding the peptide; time was needed for the droplets/aggregates to settle.

### Brightfield Microscopy Heating Analysis

Heating samples were prepared similarly to phase diagram samples, loaded on a glass well and sealed with a glass top. The glass well was loaded on a Linkam heating stage with Peltier cooling and incubated at 25 °C for 2 min, then heated at 10 °C/min to 70 °C, incubated for 2 min, and cooled down to 25 °C at 10 °C/min.

### Turbidity Analyses

All turbidity assays were performed using a BioTek H1 synergy plate reader. Here, 30 μL of each sample (n = 3 + 2 independent analyses) was loaded to a 384-well plate and measured immediately turbidity at λ = 600 nm. In the urea assay (Figure S18), the data represent a relative decrease in turbidity from T0.

### Confocal Microscopy and FRAP Analysis

All confocal micrographs were taken using a Zeiss Zen LSM 900 confocal microscope. The Cy3 collection was made using a 0.2% 561 nm laser with a pinhole of 1 AU. Samples were prepared in the same manner as mentioned above, using 75 μM of DNA, 1.11 mM of peptide, 5 mM MgCl_2_, and 50 nM of Cy3-DNA (added with nonfluorescent DNA). Samples were loaded on wells on top of a treated glass coverslip (washed with detergent and KOH) coated with Sigmacote + Pluronic F-127. All FRAP measurements used 100% of all lasers (405/488/561/640 nm) for bleaching, with 1 iteration in a 1 μm diameter circle region. The bleached sample was measured for 60 (DNA0/50/75/100) or 120 (DNAp) seconds. Analysis of the data was made upon the initial 30 (DNA0/50/75/100) or 90 (DNAp) seconds of the measurement. Raw intensity data of each time point (R1) were processed to reference point (R2) and background (R3) using the following formula:

$$IR1_{\mathrm{processed}\;\left(t\right)}=\left(IR1_{\left(t\right)}-IR3_{\left(t\right)}\right)\times \left(\frac{IR2_{\mathrm{prebleach}}-IR3_{\mathrm{prebleach}}}{IR2_{\left(t\right)}-IR3_{\left(t\right)}}\right)$$

where *I* = fluorescence intensity, and *t* = time of measurement from bleaching. Data were then normalized (0 to 100):

$$IR1_{\mathrm{normalized}\;\left(t\right)}=100\times \left(\frac{IR1_{\left(t\right)}-IR1_{\min}}{IR1_{\mathrm{prebleach}}-IR1_{\min}}\right)$$

where min = minimal intensity value throughout the experiment. The processed and normalized recovery plots were fitted to an exponential asymptotic curve using OriginLab. *t*_1/2_ was calculated as the middle *y* value of the asymptote (y = 0.5a) from the exponential equation as follows:

$$t_{1/2}=\mathrm{lo}g_{c}\left(\frac{\frac{1}{2}a}{b}\right)$$

where a is the asymptotic limit, b is the amplitude, and c is the exponential base.

### Droplet Fusion Assay

Fusion dynamics of DNA/peptide condensates were analyzed using samples containing 75 μM DNA, 11.1 mM HNP, and 5 mM MgCl_2_ in 10 mM Tris-HCl buffer pH 8. Timelapse brightfield (BF) microscopy images were taken using Olympus IX83 microscope and collected from CellSens Dimension software. After 15 min equilibration to allow condensates to settle, 7 min videos (7.6 frames s^–1^) were recorded. Samples were loaded on wells on top of a treated glass coverslip (washed with detergent and KOH) coated with Sigmacote + Pluronic F-127. All images were collected from two independent analyses (n = 20 for each DNA). Videos were processed in ImageJ, and droplet aspect ratios (AR) were measured over time by elliptical fitting:

$$AR=\frac{l_{long}}{l_{short}}$$

where $l_{\mathrm{long}}$ and $l_{\mathrm{short}}$ are the long and short axes of the ellipse. Fusion relaxation was fitted to a single-exponential decay of aspect ratio versus time to obtain the characteristic fusion time (τ), using Excel Solver:

$$AR\left(t\right)=1+\left(AR_{0}-1\right)\times \mathrm{ex}p^{\left(-\frac{t}{\tau }\right)}$$

where *AR*_0_ is the initial aspect ratio at t = 0. Mean τ values were calculated from all individual fusion events.^42^

### Small-Angle X-ray Scattering (SAXS) Analysis

Measurements were performed using an in-house X-ray scattering system, with a GeniX 3D (Xenocs) low divergence Cu Kα radiation source (X-ray wavelength, λ = 1.54 Å) and a scatterless slit setup.^43^ 2D SAXS data with a magnitude of scattering vector q covering the range of 0.002–0.3 Å^–1^ at a sample-to-detector distance of about 1 m were collected with an EIGER 1 M (Dectris) detector. The exact sample to detector position was calibrated using a silver behenate standard. Primary data processing resulting in 2D patterns reduction to 1D small-angle curves, including calibration using silver behenate as a standard and averaging of intensities, was made using the SAXSii software developed in Beck’s lab. The analysis of the SAXS 1D curves, including buffer subtraction, Guinier analysis, construction of pair distance distribution functions, dimensionless Kratky plots, and *ab initio* shape determination using dummy atom models, was performed using ATSAS software package.^44^

### SAXS Sample Preparation

Pure DNA samples for SAXS were prepared by dissolving 685.6 μg of dehydrated DNA in 100 μL of Tris-HCl buffer, pH 8. The samples with HNP were prepared similarly, where peptides were added to the buffer solution before dissolving the DNA. The measurements were conducted in a specially designed temperature-controlled flow cell (Forvis Inc.) with 0.1 °C accuracy. Before starting each measurement at a new temperature point, a waiting period of 20 min was held for the DNA systems to reach thermodynamic equilibrium. The exposure time for each sample at a given temperature was 4 h, divided into frames of 1 h each. All frames showed no noticeable changes and thus were average for better statistics.

### Computational Details

#### Coarse-Grained Molecular Dynamics Simulation Procedure

The simulation process was divided into six stages (Figure S25): MD 0, MD 1, MD 2, MD 3, MD 4, MD 5, and MD 6. MD 0 represents the all-atom (AA) structure-modeling process. MD 1 involves coarse-grained (CG) molecular dynamics simulations to sample CG models of droplets and DNAs. MD 2 simulated a mixed system including DNAs, droplets, and Mg^2+^ to sample DNA–peptide interaction modes. MD 3 focused on DNA dimer simulations induced by Mg^2+^. MD 4 performed annealing simulations (360 to 340 K) for DNAp dimers. MD 5 conducted annealing simulations (360 to 340 K) for DNAp monomers. MD 6 involved three parallel simulations that randomly packed two representative DNAp monomers from clustering DNAp monomers trajectories of MD 5. The simulation box size, trajectory duration, and system component are listed in Tables S4 and 5. All coarse-grained molecular dynamics (CGMD) simulations were performed by using the GROMACS 2022.5 software package.^45^

##### All-Atom Structures Prediction

Sequence DNA is listed in Table S1. The all-atom (AA) structures of peptides were predicted by Alphafold 3,^46^ and the AA structures (Figure S27) of DNA were built using the 3dDNA tool^46^ based on their secondary structures predicted by UNAfold.^35^

##### Coarse-Grained DNA Simulations

AA structures of DNA were coarse-grained using Martini 2.1 force field.^47,48^ The single-stranded soft (ss-soft) method was employed due to their origin from single-stranded folds and the presence of flexible regions. Charge neutrality was achieved by using Na^+^. A series of optimization steps were carried out for each system, including energy minimization within 50,000 steps under solvation conditions, followed by 1 ns of NVT and then 1 ns of NPT ensemble simulations. A time step of 20 fs was used for all the simulations. The velocity rescaling method^49^ and the Berendsen pressure coupling algorithm^49^ were employed to maintain the system at a constant temperature of 300 K and pressure of 1 bar, respectively. Bond constraints were enforced using the LINCS algorithm.^50^ Coulombic interactions were calculated with the reaction-field method, and a cutoff of 1.1 nm was used for both electrostatic and van der Waals interactions. Subsequently, a 3-μs CGMD simulation was conducted, with the same parameters as those used in the NPT ensemble simulations.

##### Coarse-Grained Droplets Simulations

We built the CG model based on the AA structure of the peptide using the Martini 2.2 force field, which includes improved parameters for peptide simulations,^47,48,51^ and assigned secondary structures using the DSSP program.^52,53^ Then, we constructed CG droplet models consisting of HNP or F5A, respectively, each of which contained 15 same-component peptides. This reflects the fact that the concentration of peptides in the experiment was 15 times the concentration of DNA. To ensure comprehensive sampling of the droplets, three distinct distributions of each droplet type were randomly generated (Figure S28), resulting in a total of six systems, all neutralized with Cl^–^. Energy minimization within 50,000 steps was carried out. A 10 ns pre-equilibration (time step: 20 fs) was conducted, during which the velocity rescale^49^ and Parrinello–Rahman^54^ methods were applied to maintain the system at 300 K and 1 bar, respectively. Subsequent to equilibration, a 14-μs CGMD simulation was performed, totaling a 42-μs CGMD simulation for each droplet system, employing the Parrinello–Rahman method for pressure coupling.^54^ All other simulation parameters were maintained consistently with those employed in the CGMD simulations of DNA.

##### Mixed Systems Simulations

Five CG mixed systems were constructed for each type of DNA, namely, F5A, HNP, Mg^2+^, F5A + Mg^2+^, and HNP + Mg^2+^. Each system was created with a box size of 18 × 18 × 18 nm^3^, containing one DNA molecule and one droplet composed of 15 peptides, with or without 37 Mg^2+^ ions. The distance between DNA and droplets was maintained around 1 nm, slightly smaller than the 1.1 nm cutoff for van der Waals interactions. A total of 10 systems were neutralized with NaCl and simulated for 3 μs, using the same scheme and force parameters as the CGMD simulations of DNA. The Lennard-Jones (LJ) parameters for Mg^2+^ were derived from the SIRAH coarse-grained force field.^55^

##### Coarse-Grained DNA Dimers Simulations

We performed CGMD simulation for DNA dimers (Figures S11–12). Each type of dimer system was created with box size of 14 × 14 × 14 nm^3^, including 2 DNA molecules, 74 Mg^2+^. All dimer systems were neutralized with NaCl and simulated for 3 μs using same parameters of CGMD simulation for 1 DNA + 37 Mg^2+^.

##### Annealing Simulations for Sampling DNAp Dimers

Two annealing simulations protocol were performed to sample DNAp dimers (Table S6). The first protocol involved annealing a system containing two DNAp molecules and 74 Mg^2+^ from 360 to 300 K, and we found a pore-like dimer, which we named DNAp dimer I (Figure S16). The second protocol first annealed a system with one DNAp molecule and 37 Mg^2+^ over the same temperature range, followed by clustering the trajectory to obtain two representative DNAp monomers (DNAp-I and DNAp-II) (Figure S34). Then, DNAp-I and DNAp-II were randomly packed together three times to generate three distinct systems, each also containing 74 Mg^2+^, which were then equilibrated at 300 K for 3 μs (Figure S13a). In the end, we obtained four DNAp dimers, including onr stable pore-like dimer, one stable head-to-tail linear dimer, and two unstable linear dimers (Figure S13b).

#### Trajectory Clustering and Representative Snapshots

We plotted representative snapshots for all simulation systems by ChimeraX,^56^ including CG models of DNAs and peptide droplets (Figure S8, Figure S32), 30 mixed systems for DNA, F5A, HNP, Mg^2+^, F5A + Mg^2+^ and HNP + Mg^2+^ (Figure S8), all DNA dimers (Figure S10), and four DNAp dimers (Figure S13).

##### Coarse-Grained Droplets

To compare the aggregation process and obtain CG models of droplets, the solvent-accessible surface area (SASA) and the radius of gyration (Rg) were calculated every 5 ns from three 14-μs trajectories for each droplet type and subsequently normalized according to eq 1.

1 $$X_{\mathrm{normalized}}={\left\{\left(X_{t}-X_{\min}\right)/\left(X_{\max}-X_{\min}\right)\right\}}_{t}^{T}$$

The SASA and Rg values were normalized with T = 8400 representing the total number of data points (SASA, Rg). The average peptide concentration increased from 85 to 120 mM over time, and the droplet aggregation process is shown in Figure S29. By mapping time values to a color gradient, the aggregation of the peptides can be clearly visualized, transitioning from purple to yellow. The degree of aggregation is highest near the point (0,0). Finally, the equilibrium concentrations of F5A and HNP were determined to be 111.82 and 111.37 mM, respectively.

A data set consisting of 3000 points was generated to construct CG droplet models. One frame was extracted every 1 ns, corresponding to the stable aggregation observed over 13–14 μs, with three independent trajectories simulated for each droplet type. Given that the SASA and Rg distributions exhibit three distinct patterns (Figure S30), the k-means clustering algorithm was applied with the number of clusters set to 3. Subsequently, proportions of each cluster and the corresponding CG droplet models were determined. The all-atom droplets were then generated using the backward.py script.^57^

##### Coarse-Grained DNA, Mixed Systems, and DNA Dimers

We sampled one frame every 1 ns over a period of 2–3 μs from each dynamic trajectory, yielding a total of 1000 frames. The CG models were generated by clustering the trajectories using the GROMOS method,^57^ with a cutoff value chosen to ensure that the largest cluster represented approximately 50% (Table S7).

##### FRET Efficiency

FRET efficiency depends not only on the distance between the donor and acceptor fluorophores, also on their mutual orientation factor (κ^2^), which is related to dipole–dipole interactions. Specifically, when κ^2^ = 0, no energy transfer occurs, regardless of the interdye distance.^39^ Under the conditions of CGMD simulations, calculating κ^2^ is challenging. However, longer fluorescence lifetimes correspond to increased steady-state fluorescence and higher energy transfer efficiency. Therefore, an empirical method for calculating FRET efficiency is defined in eq 2.

2 $$E_{\mathrm{FRET}}=\frac{\alpha }{n}\overset{n}{\underset{i}{\sum }}\frac{1}{{\mathrm{d̅}}_{ii}v_{ii}}$$

where *d̅*_*ii*_ is the average distance (nm) between *i*th bead on 5′ and 3′ terminals of DNA, being used to approximate the distance between the fluorescent groups, and *v*_*ii*_ is the standard deviation of *d̅*_*ii*_, $\frac{1}{v_{ii}}$ approximated as the degree of steady-state for energy-transfer. According to the definitions, the smaller the values of *d̅*_*ii*_ and *v*_*ii*_ are, the closer the fluorescent groups are with more stable energy transfer and thus a higher FRET efficiency. We calculated the FRET of DNA100 and DNAp systems (Figure S9, Table S9).

##### Structural Stability and Folding Behavior of DNA

Typically, in a stable evolutionary MD trajectory, smaller changes in SASA and Rg indicate greater structural stability of DNA, and smaller values of SASA and Rg indicate a greater folding of DNA. Additionally, greater structural stability also reflects the better aggregation of DNA and peptides. Therefore, the SASA and Rg values calculated were used to analyze the structural stability and folding behavior of DNAs. The structural stability of DNA was defined as the dispersion of point groups.

3 $$\mathrm{Stability}=\log\left(\frac{N_{\max}}{S}\right)$$

where *N*_max_ is the number of structures of the cluster with the most number of structures. *S* is the area of the normalized SASA and Rg from the maximum cluster.

##### Peptide–DNA Interaction Modes

To analyze the interaction mechanism between DNA with peptides, we calculated the interaction strength between the different components and the DNA sequences, based on the 2–3 μs trajectory. Since higher interaction strength indicates that more beads of the components interact with DNA, the interaction strength was defined as the number of component beads interacting with DNA. To precisely determine the interaction patterns, the cutoff for tight binding between DNA and component beads was estimated (eq 4).

4 $$d_{\mathrm{cutoff}}=\frac{1}{T}\overset{T}{\underset{t}{\sum }}d_{im}^{t},d_{im}^{t}=\frac{1}{n_{i}}\overset{n_{i}}{\underset{j}{\sum }}d_{jm}^{t}$$

where T is the frames analyzed, totaling 1000, one frame per ns within 2–3 us. *d*_*im*_^*t*^ represents the shortest distance (Å) between the *i*th deoxynucleotide and component *m* at *t* (ns), *n*_*i*_ is the number of CG beads of *i*th deoxynucleotide, *d*_*jm*_^*t*^ represents the shortest distance (Å) between *j*th bead with component *m* at *t* (ns).

For comparing the interaction patterns, the HNP + Mg^2+^ system with DNA100 was used as the reference (Figure S33). The cutoff value was estimated to be 6 Å.

#### DNA Dimers Analysis

##### Folding and Surface Characterization of DNA Dimers

We plot normalized SASA and Rg to speculate about the different folding behavior induced by Mg^2+^. Typically, DNA with larger SASA gives more contactable surface for peptides. DNA with larger Rg is more difficult to be folded by Mg^2+^. We also plotted representative snapshots for all of the DNA dimers to show their structural features.

##### Potential Energy of DNAp Dimers

To assess the relative stability of the four DNAp dimers, we calculated their potential energy distributions from stable trajectory segments (Figure S35).

##### Nonbonded Interaction Analysis of DNAp Dimers

We analyzed the nonbonded interactions (Coulombic and van der Waals) involved in the formation of four DNAp dimers. The nonbonded interaction energies of Mg^2+^-induced dimerization were calculated and compared (Figure S17, Figures S35–36), using the following formula:

5 $$E_{\mathrm{intermolecular}}^{\mathrm{nonbonded}}\left(AB\right)=E^{\mathrm{nonbonded}}\left(AB\right)-E^{\mathrm{nonbonded}}\left(A\right)-E^{\mathrm{nonbonded}}\left(B\right)$$

where A is 2 DNAp molecules, and B is coordinated Mg^2+^ between two DNAp. On the basis of their stable states, potential energy distributions, and structural features, we estimated discontinuous free energy profiles, where double slashes indicate the dimers’ distributions in conformational space may not be continuous (Figure S14).

## Supplementary Material





## Notes

Supplementary figures: DNA secondary structure predictions, CD thermal spectra, FRET emission spectra and efficiency analyses under varying conditions (Mg^2+^, peptides, dextran, urea), SAXS analyses, LLPS characterization by microscopy and turbidity, extended data on peptide–DNA interactions and control peptide comparisons, LLPS under crowding and physiological-like conditions, and visualization of DNA aggregates and condensate morphologies. Supplementary computational discussion addressing DNA dimerization mechanisms and structural ensembles by coarse-grained molecular dynamics simulations: contact maps, clustering analyses, free-energy landscapes, and interaction energy profiles. Supplementary tables provide DNA sequences, thermodynamic parameters (ΔG, Tm), FRET-derived distances, SAXS-derived structural parameters, and detailed simulation setup and clustering statistics. (PDF)
DNA random shuffle sequences FASTA (TXT)

## References

[1] Banani S. F., Lee H. O., Hyman A. A., Rosen M. K. (2017). Biomolecular Condensates:
Organizers of Cellular Biochemistry. Nat. Rev.
Mol. Cell Biol..

[2] Monette A., Niu M., Chen L., Rao S., Gorelick R. J., Mouland A. J. (2020). Pan-Retroviral
Nucleocapsid-Mediated Phase Separation Regulates Genomic RNA Positioning
and Trafficking. Cell Rep..

[3] Wang S., Dai T., Qin Z., Pan T., Chu F., Lou L., Zhang L., Yang B., Huang H., Lu H., Zhou F. (2021). Targeting Liquid-Liquid
Phase Separation of SARS-CoV-2 Nucleocapsid
Protein Promotes Innate Antiviral Immunity by Elevating MAVS Activity. Nat. Cell Biol..

[4] Jin D., Musier-Forsyth K. (2019). Role of Host TRNAs and Aminoacyl-TRNA
Synthetases in
Retroviral Replication. J. Biol. Chem..

[5] Iraci N., Tabarrini O., Santi C., Sancineto L. (2018). NCp7: Targeting
a Multitask Protein for next-Generation Anti-HIV Drug Development
Part 2. Noncovalent Inhibitors and Nucleic Acid Binders. Drug Discovery Today.

[6] Cao S., Ivanov T., Heuer J., Ferguson C. T. J., Landfester K., Caire da Silva L. (2024). Dipeptide
Coacervates as Artificial Membraneless Organelles
for Bioorthogonal Catalysis. Nat. Commun..

[7] Kang H., Yoo J., Sohn B.-K., Lee S.-W., Lee H. S., Ma W., Kee J.-M., Aksimentiev A., Kim H. (2018). Sequence-Dependent
DNA Condensation as a Driving Force of DNA Phase Separation. Nucleic Acids Res..

[8] Baruch
Leshem A., Gaash D., Lampel A. (2026). Design and Applications
of Synthetic Biomolecular Condensates. Nat.
Nanotechnol..

[9] Roden C., Gladfelter A. S. (2021). RNA Contributions to the Form and
Function of Biomolecular
Condensates. Nat. Rev. Mol. Cell Biol..

[10] Nguyen H. T., Hori N., Thirumalai D. (2022). Condensates in RNA Repeat Sequences
Are Heterogeneously Organized and Exhibit Reptation Dynamics. Nat. Chem..

[11] Guo W., Ji D., Kinghorn A. B., Chen F., Pan Y., Li X., Li Q., Huck W. T. S., Kwok C. K., Shum H. C. (2023). Tuning
Material
States and Functionalities of G-Quadruplex-Modulated RNA-Peptide Condensates. J. Am. Chem. Soc..

[12] Liu W., Deng J., Song S., Sethi S., Walther A. (2024). A Facile DNA
Coacervate Platform for Engineering Wetting, Engulfment, Fusion and
Transient Behavior. Commun. Chem..

[13] Aumiller W. M., Keating C. D. (2016). Phosphorylation-Mediated
RNA/Peptide Complex Coacervation
as a Model for Intracellular Liquid Organelles. Nat. Chem..

[14] Alshareedah I., Moosa M. M., Pham M., Potoyan D. A., Banerjee P. R. (2021). Programmable
Viscoelasticity in Protein-RNA Condensates with Disordered Sticker-Spacer
Polypeptides. Nat. Commun..

[15] Wadsworth G. M., Zahurancik W. J., Zeng X., Pullara P., Lai L. B., Sidharthan V., Pappu R. V., Gopalan V., Banerjee P. R. (2023). RNAs Undergo
Phase Transitions with Lower Critical Solution Temperatures. Nat. Chem..

[16] Netzer A., Baruch Leshem A., Veretnik S., Edelstein I., Lampel A. (2024). Regulation of Peptide
Liquid-Liquid Phase Separation
by Aromatic Amino Acid Composition. Small.

[17] Netzer A., Katzir I., Baruch Leshem A., Weitman M., Lampel A. (2023). Emergent Properties
of Melanin-Inspired Peptide/RNA Condensates. Proc. Natl. Acad. Sci. U. S. A..

[18] Choi S., Meyer M. O., Bevilacqua P. C., Keating C. D. (2022). Phase-Specific RNA
Accumulation and Duplex Thermodynamics in Multiphase Coacervate Models
for Membraneless Organelles. Nat. Chem..

[19] Vieregg J. R., Lueckheide M., Marciel A. B., Leon L., Bologna A. J., Rivera J. R., Tirrell M. V. (2018). Oligonucleotide-Peptide Complexes:
Phase Control by Hybridization. J. Am. Chem.
Soc..

[20] Lebold K. M., Best R. B. (2022). Tuning Formation of Protein-DNA Coacervates by Sequence
and Environment. J. Phys. Chem. B.

[21] Mimura M., Tomita S., Shinkai Y., Hosokai T., Kumeta H., Saio T., Shiraki K., Kurita R. (2021). Quadruplex Folding
Promotes the Condensation of Linker Histones and DNAs via Liquid-Liquid
Phase Separation. J. Am. Chem. Soc..

[22] Bian Y., Lv F., Pan H., Ren W., Zhang W., Wang Y., Cao Y., Li W., Wang W. (2024). Fusion Dynamics and Size-Dependence
of Droplet Microstructure in SsDNA-Mediated Protein Phase Separation. JACS Au.

[23] Green C. M., Sementa D., Mathur D., Melinger J. S., Deshpande P., Elbaum-Garfinkle S., Medintz I. L., Ulijn R. V., Díaz S. A. (2024). Sequestration
within Peptide Coacervates Improves the Fluorescence Intensity, Kinetics,
and Limits of Detection of Dye-Based DNA Biosensors. Commun. Chem..

[24] Samanta A., Baranda Pellejero L., Masukawa M., Walther A. (2024). DNA-Empowered
Synthetic
Cells as Minimalistic Life Forms. Nat. Rev.
Chem..

[25] Seeman N. C., Sleiman H. F. (2018). DNA Nanotechnology. Nat. Rev.
Mater..

[26] Sato Y., Sakamoto T., Takinoue M. (2020). Sequence-Based Engineering of Dynamic
Functions of Micrometer-Sized DNA Droplets. Sci. Adv..

[27] Biffi S., Cerbino R., Bomboi F., Paraboschi E. M., Asselta R., Sciortino F., Bellini T. (2013). Phase Behavior and
Critical Activated Dynamics of Limited-Valence DNA Nanostars. Proc. Natl. Acad. Sci. U. S. A..

[28] Jeon B.-J., Nguyen D. T., Abraham G. R., Conrad N., Fygenson D. K., Saleh O. A. (2018). Salt-Dependent Properties
of a Coacervate-like, Self-Assembled
DNA Liquid. Soft Matter.

[29] Merindol R., Loescher S., Samanta A., Walther A. (2018). Pathway-Controlled
Formation of Mesostructured All-DNA Colloids and Superstructures. Nat. Nanotechnol..

[30] Woodson S. A., Panja S., Santiago-Frangos A. (2018). Proteins That
Chaperone RNA Regulation. Microbiol. Spectr..

[31] Chau B.-A., Chen V., Cochrane A. W., Parent L. J., Mouland A. J. (2023). Liquid-Liquid
Phase Separation of Nucleocapsid Proteins during SARS-CoV-2 and HIV-1
Replication. Cell Rep..

[32] Savastano A., Ibáñez de
Opakua A., Rankovic M., Zweckstetter M. (2020). Nucleocapsid
Protein of SARS-CoV-2 Phase Separates into RNA-Rich Polymerase-Containing
Condensates. Nat. Commun..

[33] Katzir I., Haimov E., Lampel A. (2022). Tuning the
Dynamics of Viral-Factories-Inspired
Compartments Formed by Peptide-RNA Liquid-Liquid Phase Separation. Adv. Mater..

[34] Meyer M. O., Yamagami R., Choi S., Keating C. D., Bevilacqua P. C. (2023). RNA Folding
Studies inside Peptide-Rich Droplets Reveal Roles of Modified Nucleosides
at the Origin of Life. Sci. Adv..

[35] Markham N. R., Zuker M. (2008). UNAFold: Software for
Nucleic Acid Folding and Hybridization. Methods
Mol. Biol..

[36] Luan B., Aksimentiev A. (2008). DNA Attraction in Monovalent and Divalent Electrolytes. J. Am. Chem. Soc..

[37] Mitra M., Wang W., Vo M.-N., Rouzina I., Barany G., Musier-Forsyth K. (2013). The N-Terminal
Zinc Finger and Flanking Basic Domains
Represent the Minimal Region of the Human Immunodeficiency Virus Type-1
Nucleocapsid Protein for Targeting Chaperone Function. Biochemistry.

[38] Rong L., Russell R. S., Hu J., Guan Y., Kleiman L., Liang C., Wainberg M. A. (2001). Hydrophobic
Amino Acids in the Human
Immunodeficiency Virus Type 1 P2 and Nucleocapsid Proteins Can Contribute
to the Rescue of Deleted Viral RNA Packaging Signals. J. Virol..

[39] Kyrychenko A., Rodnin M. V., Ghatak C., Ladokhin A. S. (2017). Joint Refinement
of FRET Measurements Using Spectroscopic and Computational Tools. Anal. Biochem..

[40] Mouhand A., Pasi M., Catala M., Zargarian L., Belfetmi A., Barraud P., Mauffret O., Tisné C. (2020). Overview of
the Nucleic-Acid Binding Properties of the HIV-1 Nucleocapsid Protein
in Its Different Maturation States. Viruses.

[41] Lambert T. J. (2019). FPbase:
A Community-Editable Fluorescent Protein Database. Nat. Methods.

[42] Brangwynne C. P., Mitchison T. J., Hyman A. A. (2011). Active Liquid-like
Behavior of Nucleoli
Determines Their Size and Shape in Xenopus Laevis Oocytes. Proc. Natl. Acad. Sci. U. S. A..

[43] Li Y., Beck R., Huang T., Choi M. C., Divinagracia M. (2008). Scatterless
Hybrid Metal–Single-Crystal Slit for Small-Angle X-Ray Scattering
and High-Resolution X-Ray Diffraction. J. Appl.
Crystallogr..

[44] Manalastas-Cantos K., Konarev P. V., Hajizadeh N. R., Kikhney A. G., Petoukhov M. V., Molodenskiy D. S., Panjkovich A., Mertens H. D. T., Gruzinov A., Borges C., Jeffries C. M., Svergun D. I., Franke D. (2021). ATSAS 3.0:
Expanded Functionality and New Tools for Small-Angle Scattering Data
Analysis. J. Appl. Crystallogr..

[45] Abraham M. J., Murtola T., Schulz R., Páll S., Smith J. C., Hess B., Lindahl E. (2015). GROMACS: High
Performance
Molecular Simulations through Multi-Level Parallelism from Laptops
to Supercomputers. SoftwareX.

[46] Abramson J., Adler J., Dunger J., Evans R., Green T., Pritzel A., Ronneberger O., Willmore L., Ballard A. J., Bambrick J., Bodenstein S. W., Evans D. A., Hung C.-C., O’Neill M., Reiman D., Tunyasuvunakool K., Wu Z., Žemgulytė A., Arvaniti E., Beattie C., Bertolli O., Bridgland A., Cherepanov A., Congreve M., Cowen-Rivers A. I., Cowie A., Figurnov M., Fuchs F. B., Gladman H., Jain R., Khan Y. A., Low C. M. R., Perlin K., Potapenko A., Savy P., Singh S., Stecula A., Thillaisundaram A., Tong C., Yakneen S., Zhong E. D., Zielinski M., Žídek A., Bapst V., Kohli P., Jaderberg M., Hassabis D., Jumper J. M. (2024). Accurate Structure
Prediction of
Biomolecular Interactions with AlphaFold 3. Nature.

[47] Marrink S. J., Risselada H. J., Yefimov S., Tieleman D. P., de Vries A. H. (2007). The MARTINI
Force Field: Coarse Grained Model for Biomolecular Simulations. J. Phys. Chem. B.

[48] Monticelli L., Kandasamy S. K., Periole X., Larson R. G., Tieleman D. P., Marrink S.-J. (2008). The MARTINI
Coarse-Grained Force Field: Extension to
Proteins. J. Chem. Theory Comput..

[49] Bussi G., Donadio D., Parrinello M. (2007). Canonical
Sampling through Velocity
Rescaling. J. Chem. Phys..

[50] Hess B., Bekker H., Berendsen H. J. C., Fraaije J. G. E. M. (1997). LINCS: A linear
constraint solver for molecular simulations. Journal of Computational Chemistry.

[51] de
Jong D. H., Singh G., Bennett W. F. D., Arnarez C., Wassenaar T. A., Schäfer L. V., Periole X., Tieleman D. P., Marrink S. J. (2013). Improved Parameters for the MARTINI Coarse-Grained
Protein Force Field. J. Chem. Theory Comput..

[52] Touw W. G., Baakman C., Black J., te Beek T. A. H., Krieger E., Joosten R. P., Vriend G. (2015). A Series of
PDB-Related Databanks
for Everyday Needs. Nucleic Acids Res..

[53] Kabsch W., Sander C. (1983). Dictionary of Protein Secondary Structure: Pattern
Recognition of Hydrogen-Bonded and Geometrical Features. Biopolymers.

[54] Parrinello M., Rahman A. (1981). Polymorphic Transitions
in Single Crystals: A New Molecular
Dynamics Method. J. Appl. Phys..

[55] Klein F., Cáceres D., Carrasco M. A., Tapia J. C., Caballero J., Alzate-Morales J., Pantano S. (2020). Coarse-Grained Parameters for Divalent
Cations within the SIRAH Force Field. J. Chem.
Inf. Model..

[56] Meng E. C., Goddard T. D., Pettersen E. F., Couch G. S., Pearson Z. J., Morris J. H., Ferrin T. E. (2023). UCSF ChimeraX:
Tools for Structure
Building and Analysis. Protein Sci..

[57] Wassenaar T. A., Pluhackova K., Böckmann R. A., Marrink S. J., Tieleman D. P. (2014). Going Backward:
A Flexible Geometric Approach to Reverse Transformation from Coarse
Grained to Atomistic Models. J. Chem. Theory
Comput..

