# Supplementary material for: Programmable DNA Folding Modulates Phase Behavior and Dynamics of DNA/Peptide Condensates
Source: ACS Nano. 2026 May 13;20(20):14813–27. doi: 10.1021/acsnano.6c03646 (PMC13218041; doi:10.1021/acsnano.6c03646)
Supplement: Supplementary file 1 [file nn6c03646_si_001.pdf]

Supporting Information

Programmable DNA folding modulates phase behavior and dynamics of DNA/peptide condensates

Itai Katzir[1^], Yanbing Wen[2^], Inbal Razi[1], Vadim Skoi[3], Roy Beck[3-6], Hao Dong[2]*, and Ayala Lampel[1,4,5,7]*

[1]Shmunis School of Biomedicine and Cancer Research, George S. Wise Faculty of Life Sciences, Tel Aviv University, Tel Aviv 6997801 Israel.

[2]State Key Laboratory of Analytical Chemistry for Life Science, Kuang Yaming Honors School, Chemistry and Biomedicine Innovation Center (Chem-BIC), ChemBioMed Interdisciplinary Research Center at Nanjing University, & Institute for Brain Sciences, Nanjing University, Nanjing 210023, China.

[3]School of Physics and Astronomy, Tel Aviv University, Tel Aviv 6997801, Israel.

[4]Center for the Physics and Chemistry of Living Systems Tel Aviv University, Tel Aviv 6997801, Israel.

[5]Center for Nanoscience and Nanotechnology Tel Aviv University, Tel Aviv 6997801, Israel.

[6]Sagol School of Neuroscience, Tel Aviv University, Tel Aviv 6997801, Israel.

[7]Division Polymer Biomaterials Science, Leibniz Institute of Polymer Research Dresden, 01069 Dresden, Germany.

^Authors have equally contributed to the work

*co-corresponding authors

Supplementary Figures

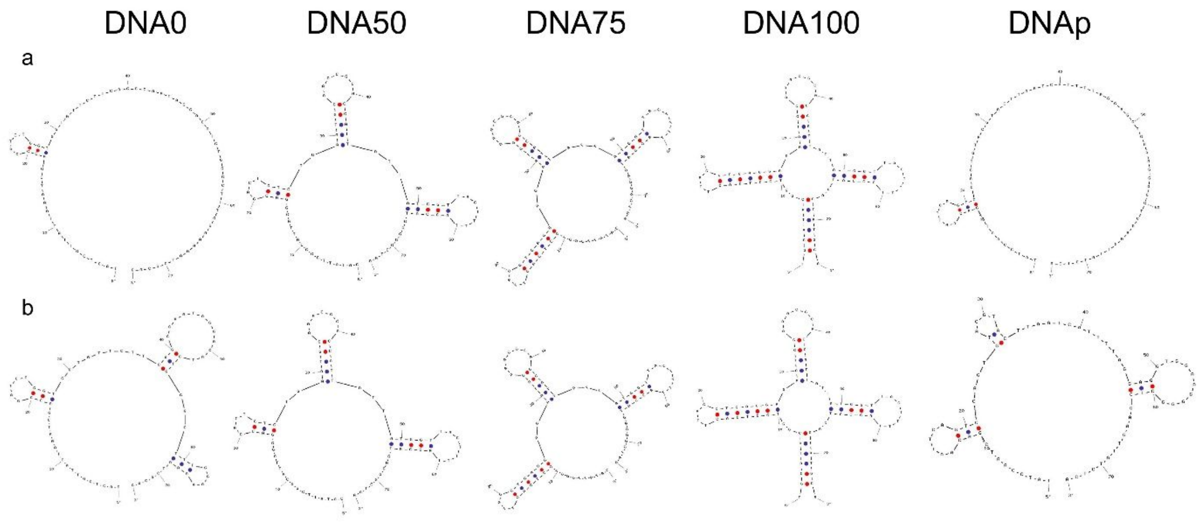

Figure S1. DNA secondary structure prediction by UNAfold. Secondary structures of all DNA sequences in 10 mM Tris buffer without (**a**) or with (**b**) 10 mM Mg^{2+}. **b**. DNA100: stem (bases: 2-7 & 68-73), hairpin1 (bases: 10-27), hairpin2 (base: 29-45), hairpin3 (bases: 49-65) and flexible regions (bases: 8-9, 28, 46-48 & 66-67). DNA75: hairpin 1,2&3, not including stem. DNA50: hairpin 2&3, not including stem and hairpin 1. DNA0: hairpin 1 (18-28), hairpin 2 (38-55) and hairpin 3 (59-68). DNAp: hairpin 1 (10-21) in both outputs, hairpin 2 (26-33) that is only present with Mg^{2+}, hairpin 3 (bases: 47-62) also present only with Mg^{2+}.

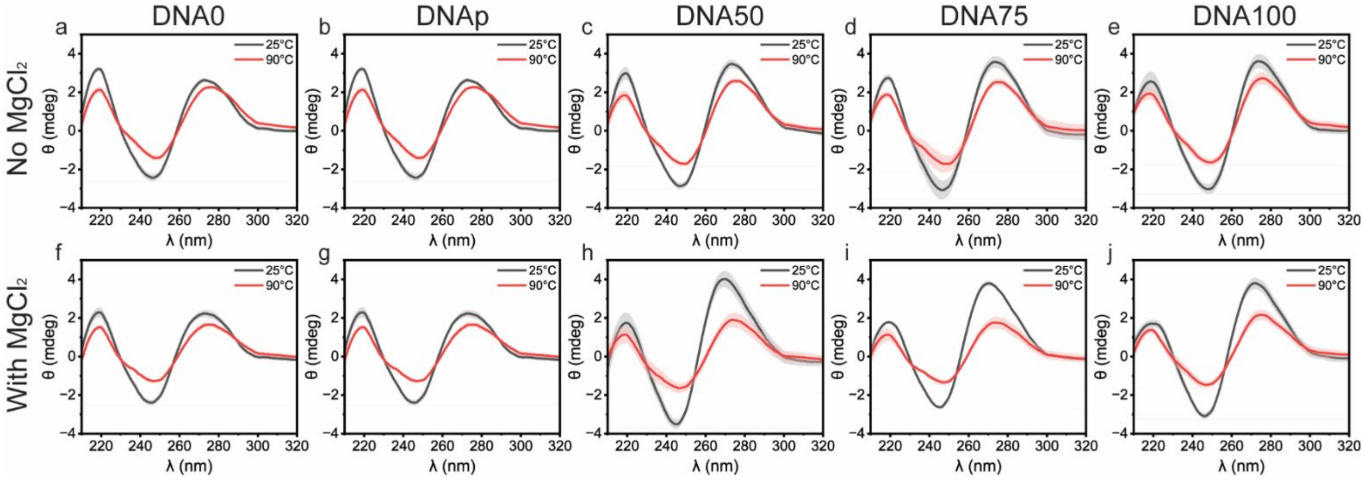

Figure S2. CD Spectra of DNAs at varying temperatures. a-e. CD spectra of all DNAs in Tris-HCl buffer at 25 °C (dark grey) or 90 °C (red). **f-j**. CD spectra of all DNAs in Tris-HCl buffer with 1.5 mM $MgCl_2$ at 1:300 DNA:$MgCl_2$ molar ratio at 25 °C or 90 °C. DNA concentration in all samples is 5 μM.

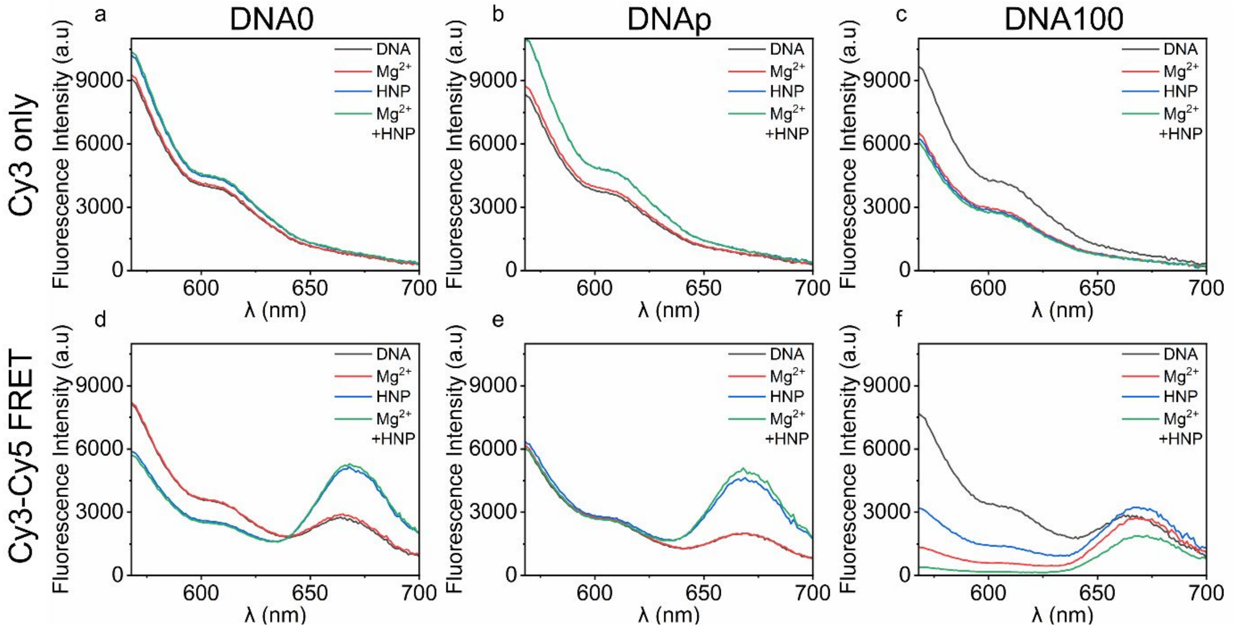

Figure S3. DNA-$MgCl_2$-HNP FRET emission spectra. a-c. Emission spectra of (**a**) Cy3-DNA0 (**b**) Cy3-DNAp (**c**) Cy3-DNA100. **d-f**. Emission spectra of (**d**) Cy3-DNA0-Cy5, (**e**) Cy3-DNAp-Cy5, (**f**) Cy3-DNA100-Cy5. Excitation wavelength is λ_{ex}=540 nm.

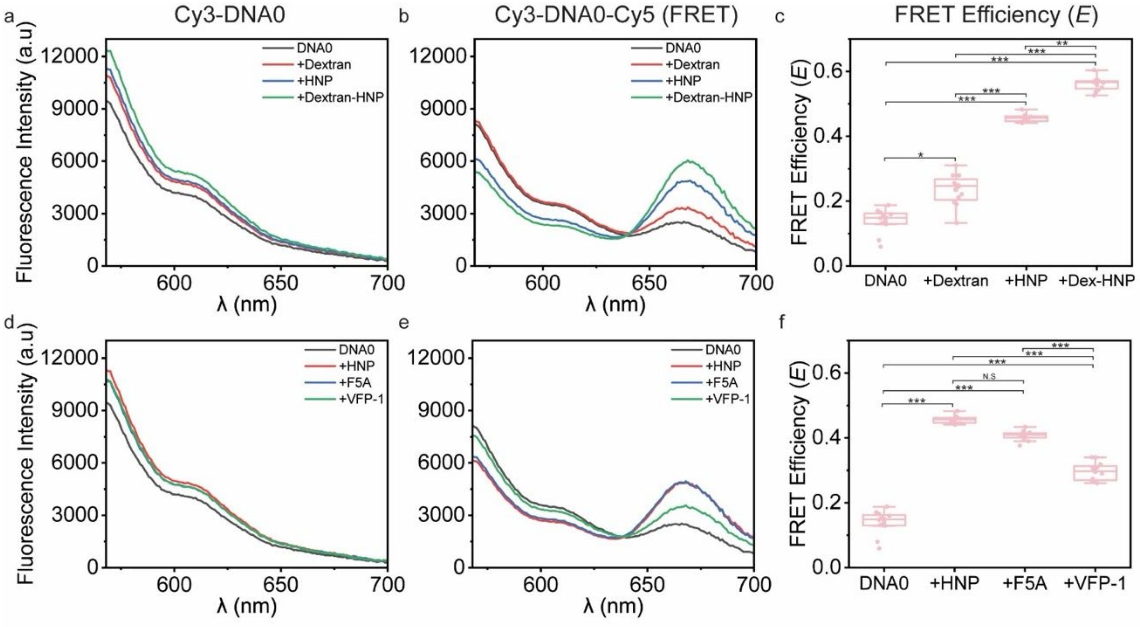

Figure S4. DNA0 FRET analyses. **a-c**. FRET in presence of 20 w/v% dextran and HNP. **a-b**. Cy3-DNA0 (**a**) and Cy3-DNA0-Cy5 FRET pair (**b**) emission spectra. **c**. FRET efficiency (*E*) box plots of corresponding FRET samples. **d-f**. FRET of DNA0 with different cationic peptides. **d-e**. Cy3-DNA0 (**d**) and Cy3-DNA0-Cy5 FRET pair (**e**) emission spectra either in the absence or presence of different cationic peptides. **f**. FRET efficiency (*E*) box plots of corresponding FRET samples. Excitation wavelength is λ_{ex}=540 nm. Boxes showing n=4 measurements from three independent analyses. Statistics are derived from one-way ANOVA test with Tukey's post hoc test.

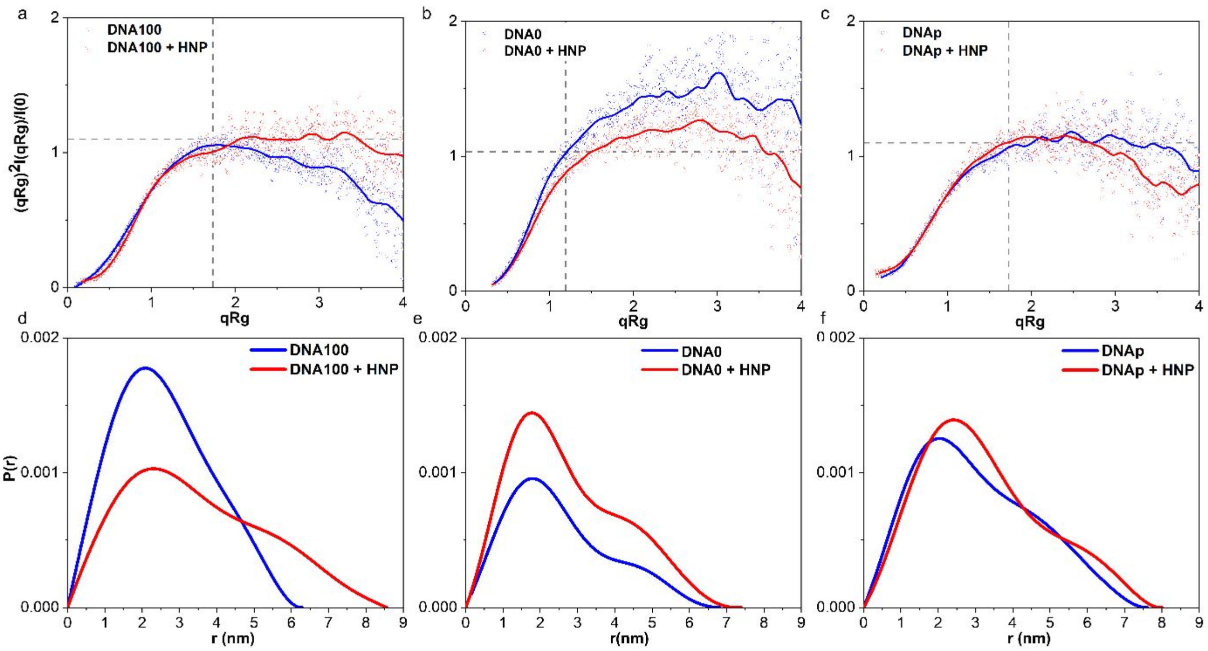

Figure S5. Dimensionless Kratky plots for DNA100 (**a**) and DNA0 (**b**) and DNAp (**c**) either alone (blue) or in the presence of HNP peptide (red) at 25 °C. The experimental data is shown as dots, the solid lines represent the smoothed Kratky plots to improve readability. The dashed lines are drawn vertically at the abscissa value $qRg=\sqrt{3} \approx 1.73$ and horizontally at the ordinate value $3/e \approx 1.1$ for visual guidance. Pair distance distribution functions for DNA100 (**d**) and DNA0 (**e**) and DNAp (**f**) either alone (blue) or in the presence of HNP peptide (red) with and without HNP.

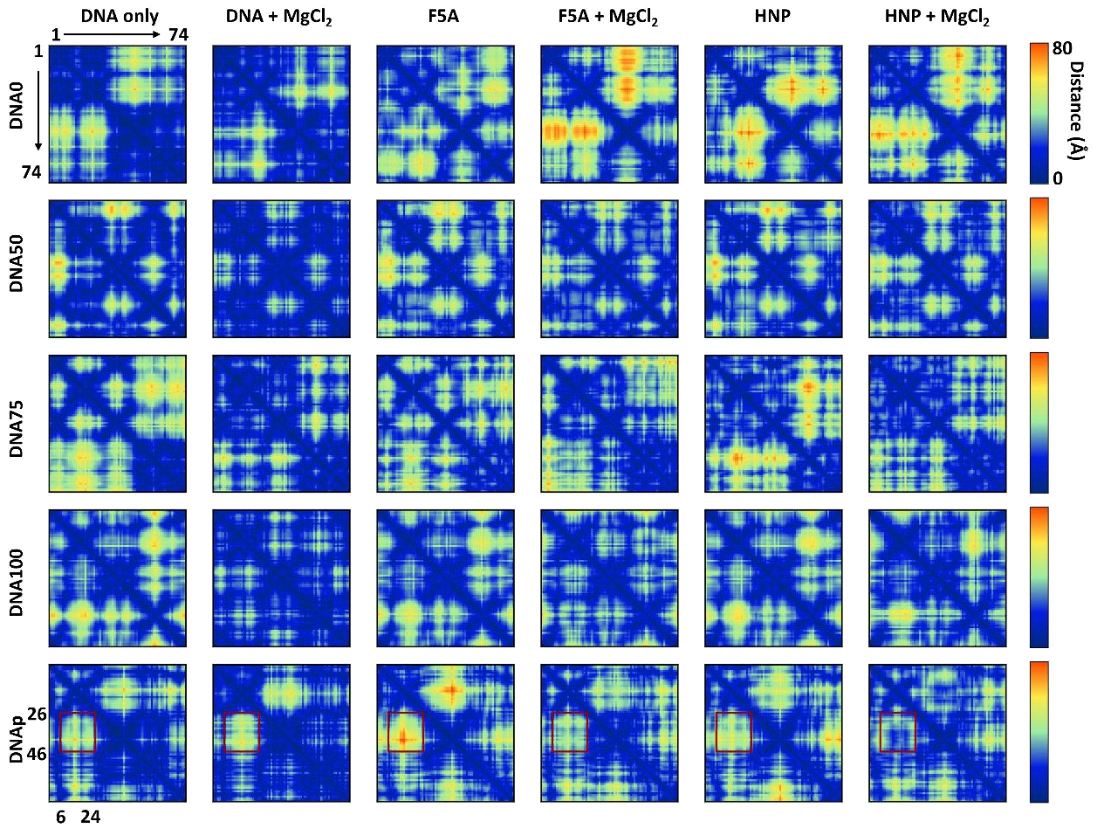

Figure S6. Contact distance matrix of DNA. The pairwise distances between coarse-grained SC2 beads of DNA deoxynucleotides are represented as a heatmap (color scale: 0 to 80 Å). The red boxes highlight contacts involving residues 6–24 and 26–46 of the DNAp sequence, with residues 26–33 corresponding to the palindromic region.

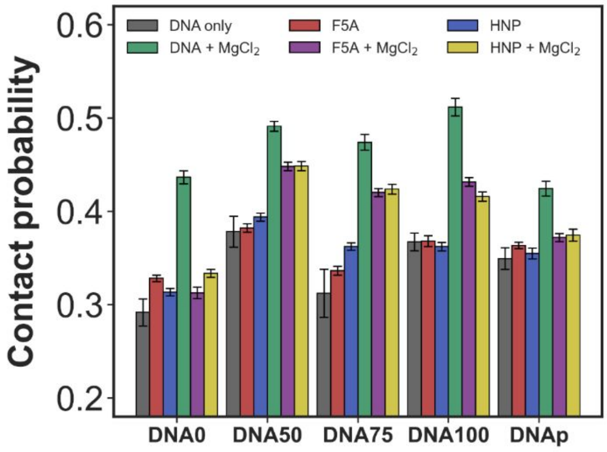

Figure S7. Contact probability of DNA residues across all simulated systems. A contact is defined when the corresponding distance matrix element is less than twice the van der Waals interaction distance (11 Å). The contact probabilities are obtained by averaging over the most representative conformational ensemble after trajectory clustering.

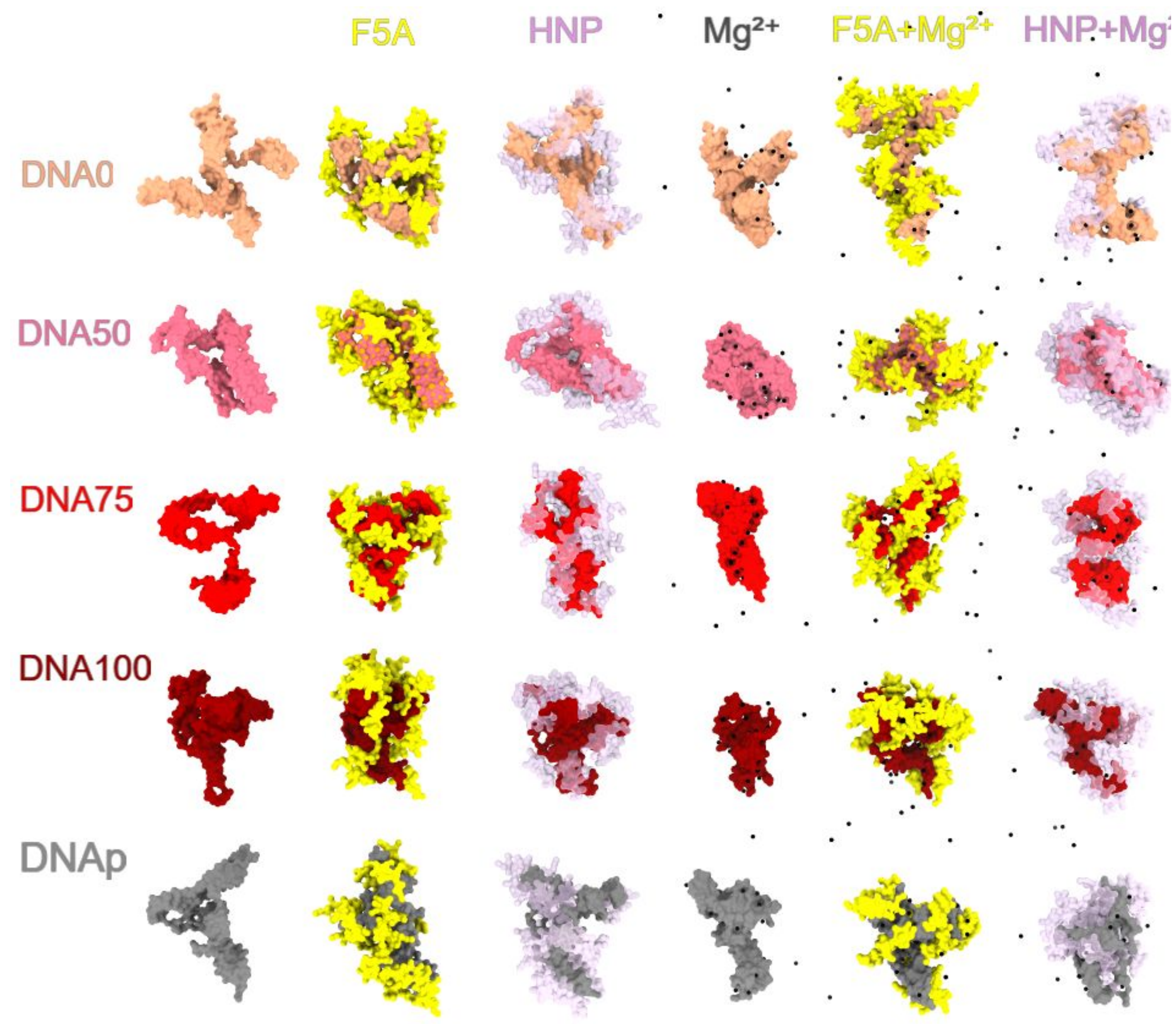

Figure S8. Representative snapshots of all simulated systems, including DNA-only and DNA-substrate complexes. Color-code: DNA0 (light red), DNA50 (orange), DNA75 (red), DNA100 (dark red), and DNAp (gray), peptides (F5A: yellow, HNP: pink), and Mg^{2+} ions (black dots) are rendered in contrasting colors for clarity.

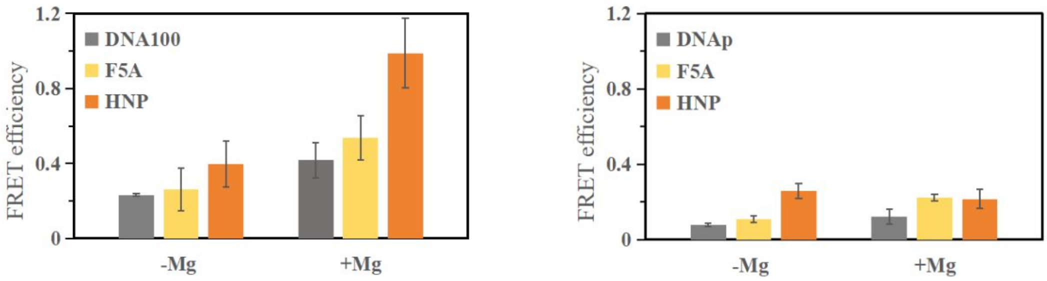

Figure S9. FRET efficiency for DNA100 (left) and DNAp (right) predicted with CGMD simulations.

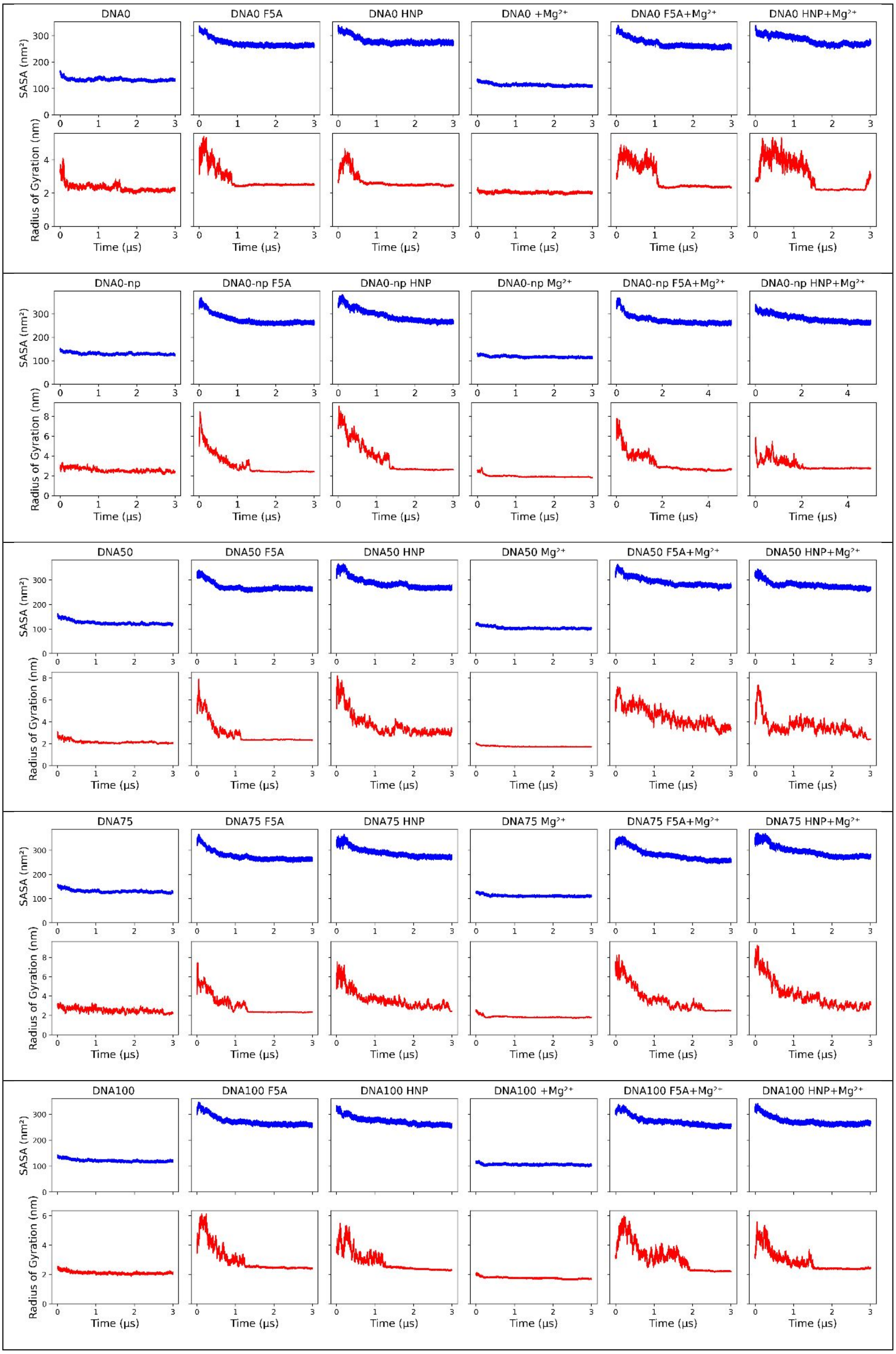

Figure S10. The calculated SASA and Rg of all systems from CGMD simulations.

Supplementary Computational Discussion

DNAp dimerization and structural characterization. To explore potential DNA-DNA self-association mechanisms, we systematically sampled dimers for all DNA constructs at 300 K in the presence of Mg^{2+} ions. Analysis of the resulting ensembles revealed distributions of Rg and SASA, together with representative conformations (Fig. S9-S10). Among the constructs examined, DNAp dimers exhibited the largest Rg and highest SASA, indicating an extended, solvent-exposed conformations. This behavior is attributed to the palindromic architecture of DNAp, which promotes rod-like arrangements and provides increased surface area for peptide association.

To further probe DNAp dimerization mechanisms, additional dimers were sampled using annealing simulations (Fig. S11). Free-energy landscapes were estimated based on dimerization patterns, potential energy distributions, and persistence times of stable conformations (Fig. S12-S14). This analysis identified four recurrent dimer configurations, including two stable states (dimers I, III) and two metastable linear arrangements (dimers II & IV). Two dominant configurations, a pore-like dimer (dimer I) and a head-to-tail linear dimer (dimer III), exhibited enhanced peptide aggregation propensity in simulations. The pore-like configuration contains a central channel on the order of 10-20 Å, whereas the head-to-tail arrangement is stabilized by Mg^{2+} coordination between palindromic regions and 3' DNAp end phosphate groups, suggesting a possible route toward extended assemblies. Analysis of non-bonded interaction energies indicates that electrostatic interactions dominate DNAp dimer stabilization, with Mg^{2+} coordination playing a central role, while van der Waals contributions are comparatively minor (Fig. S15). Together, these simulations suggest that DNAp self-recognition can generate extended, peptide-accessible interfaces under Mg^{2+}-rich conditions, potentially enhancing multivalent interactions relevant to condensate formation.

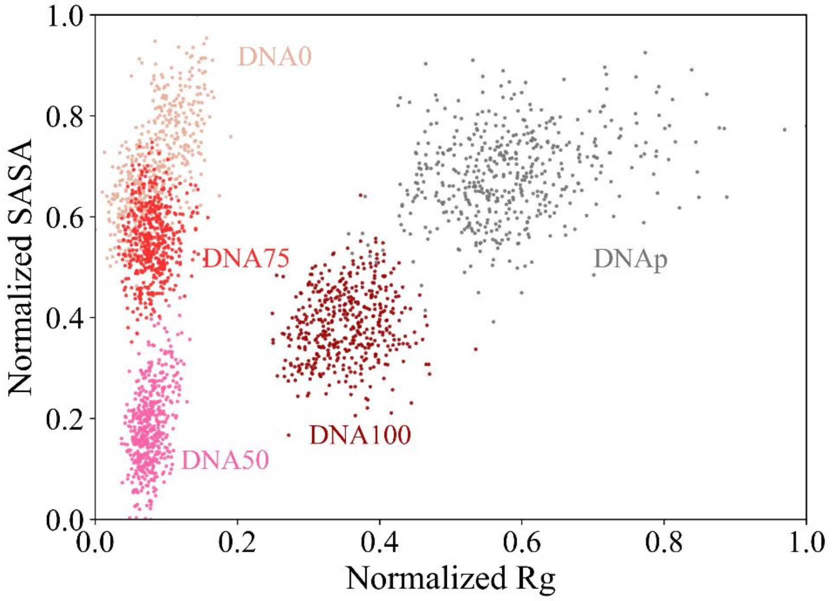

Figure S11. Scatter plot of normalized Rg versus normalized SASA for DNA dimers. DNAp dimer exhibits the largest Rg and SASA, indicating an extended and solvent-exposed conformation.

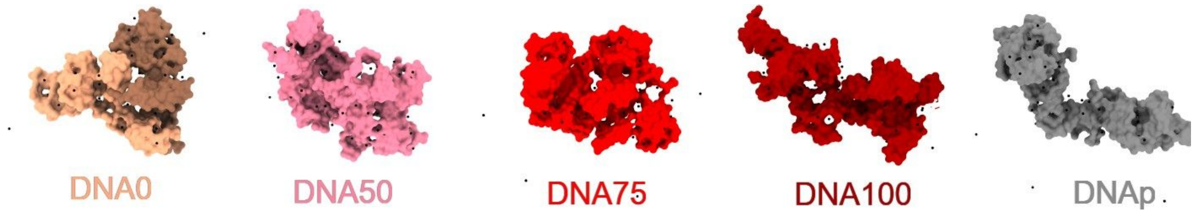

Figure S12. Representative snapshots of DNA dimers.

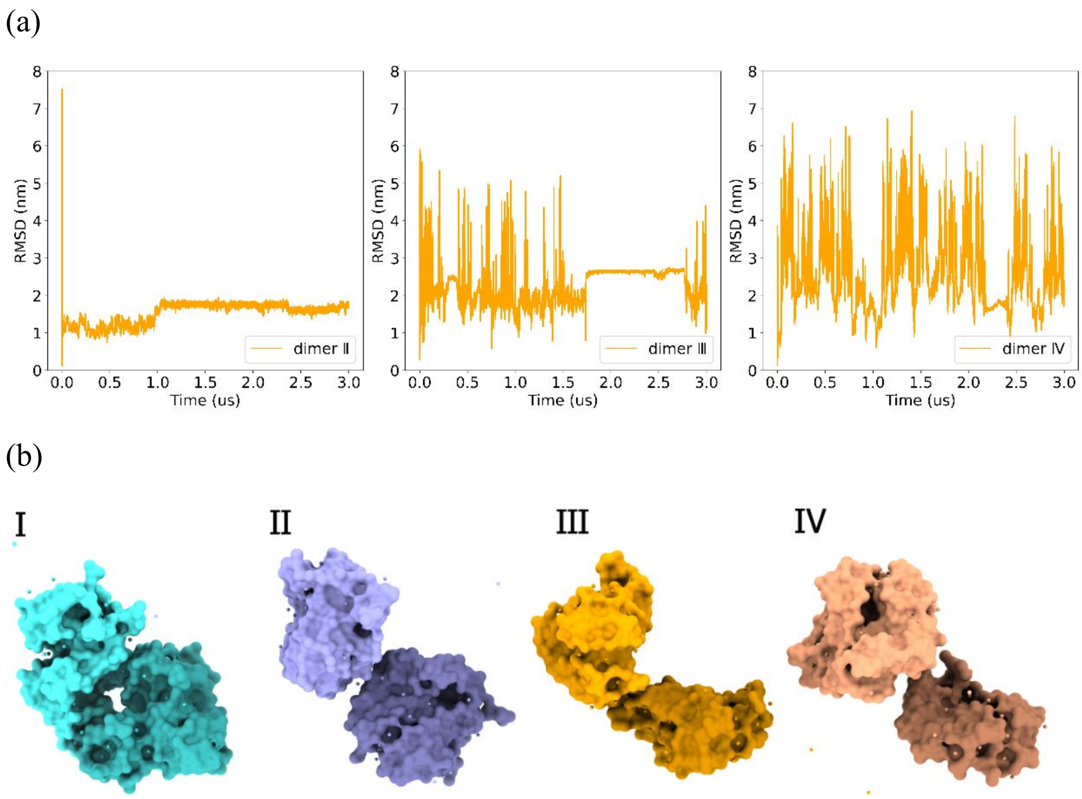

Figure S13. DNAp dimers. (a) Three parallel simulations of DNAp Dimers (300 K). (b) Representative configurations of DNAp dimers generated from simulations.

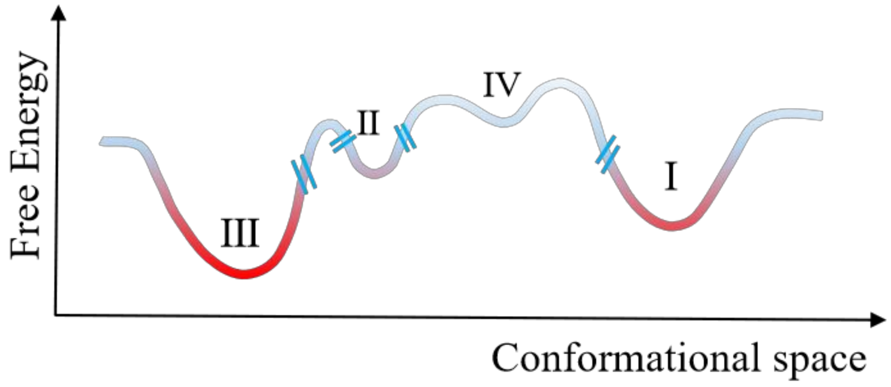

Figure S14. Schematic illustration of the free energy landscape for different states (Ⅰ, Ⅱ, Ⅲ and Ⅳ) of DNAp aggregates. Truncation indicates potential conformational discontinuity.

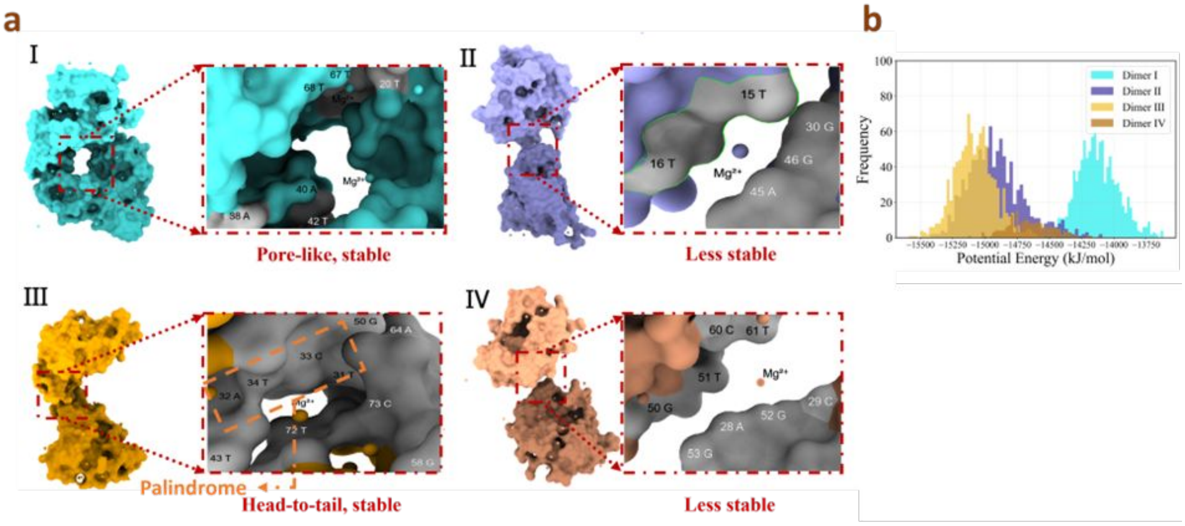

Figure S15. DNAp dimer conformations. (a) Structures of four DNAp dimers (Ⅰ: pore-type with a diameter of approximately 10–20 Å; Ⅱ–Ⅳ: linear-type with different Mg^{2+}-phosphate coordination modes) are displayed, with insets showing key coordination sites (e.g., 68 T-Mg^{2+}-20 T in dimer Ⅰ, T34-Mg^{2+}-T72 in dimer Ⅲ). (b) Potential energy distributions reveal mean ± variance values: dimer Ⅰ (-1.41×10^4 kJ/mol, 2.66×10^4 (kJ/mol)^2), dimer Ⅱ (-1.49×10^4, 4.93×10^4), dimer Ⅲ (-1.51×10^4, 2.62×10^4), and dimer Ⅳ (-1.46×10^4, 3.23×10^4).

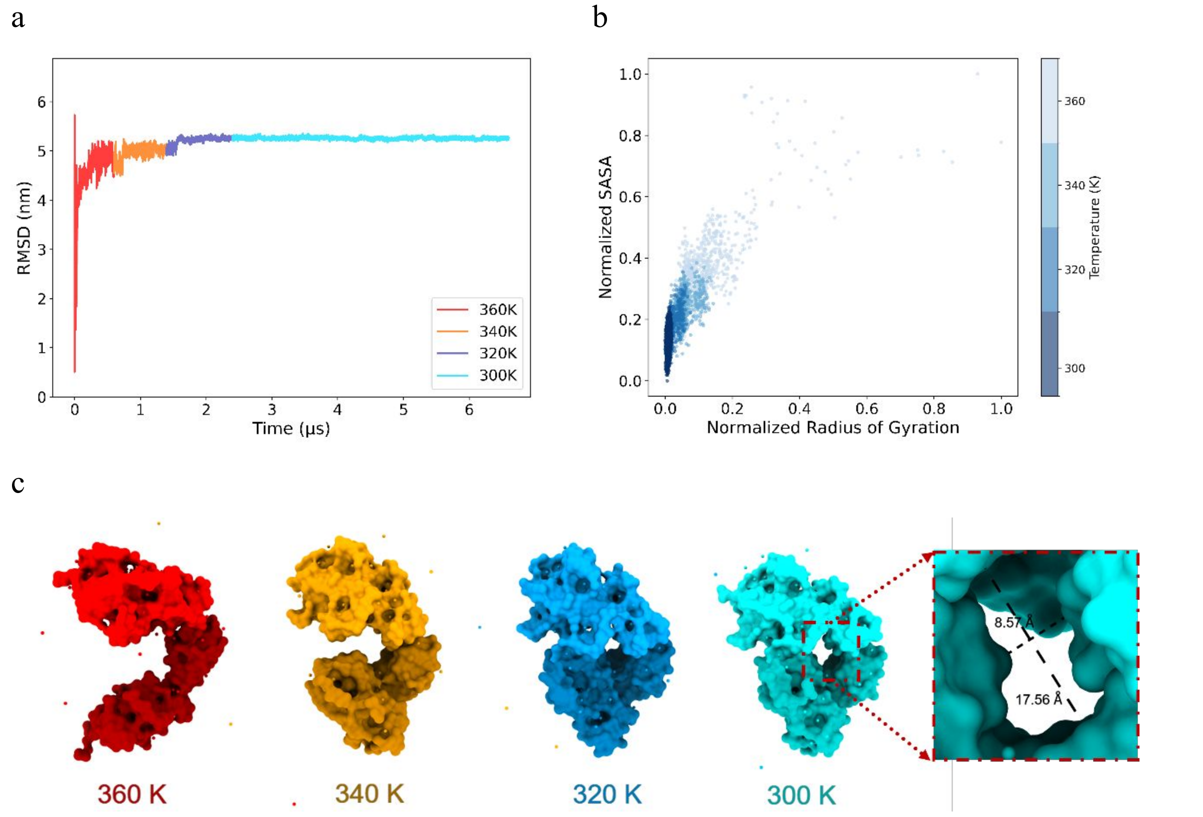

Figure S16. Structural ensemble of the DNAp dimer generated using the simulated annealing method. (a) RMSD over time at different temperatures (360 K, 340 K, 320 K and 300 K). (b-c) The formation process of DNAp pore-like dimer. At 300 K, the dimer forms a distinct central pore with a diameter of approximately 10–20 Å, as highlighted in the magnified view on the right, where specific measurements of 8.57 Å and 17.56 Å are annotated.

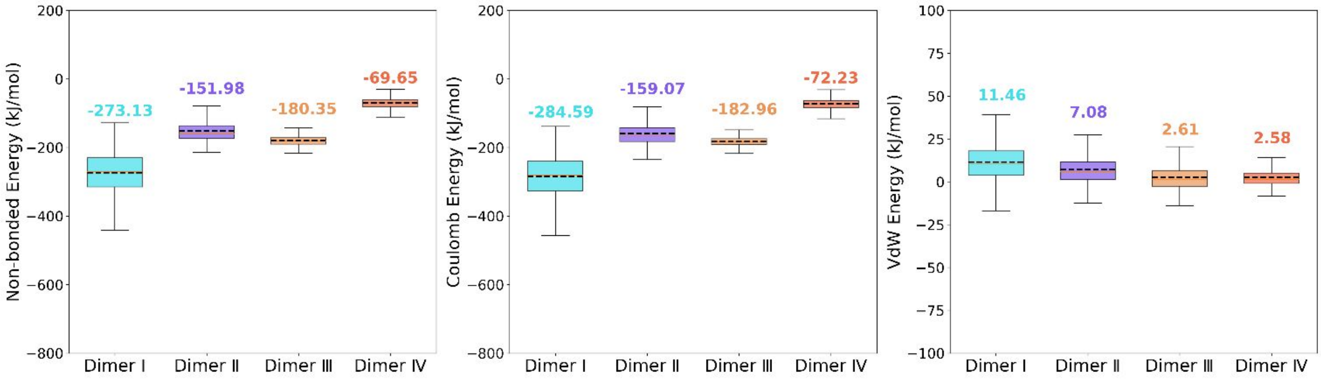

Figure. S17. Non-bonded interaction energies during DNAp dimers formation. Energy distributions: Coulomb energy dominates dimer stabilization (-72.23 to -284.59 kJ/mol) while van der Waals energy increases moderately (2.58 to 11.46 kJ/mol), resulting in net negative total non-bonded energy (-69.65 to -273.13 kJ/mol).

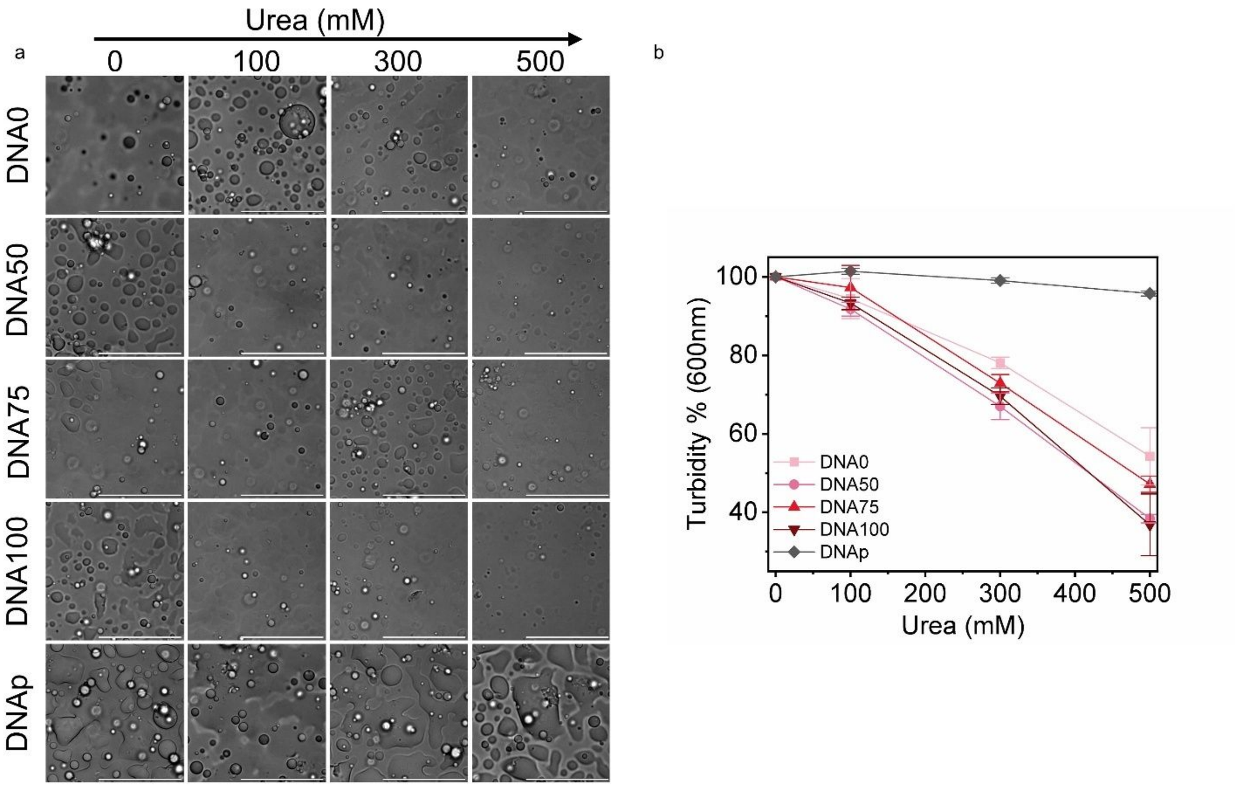

Figure S18. Stability of DNA-HNP condensates in urea. a. Microscopy images of DNA-HNP condensates at increasing concentrations of urea (mM). **b**. Relative decrease in turbidity (λ=600 nm) of LLPS samples at increasing urea concentration. All samples are made with 75 μM DNA and 1.11 mM HNP at 1:1 charge ratio, in Tris-HCl (10mM pH 8) containing 5 mM $MgCl_2$. Values represent averages of n=3-4 from 2 independent measurements, error bars represent S.D. Scale bars=50 μm.

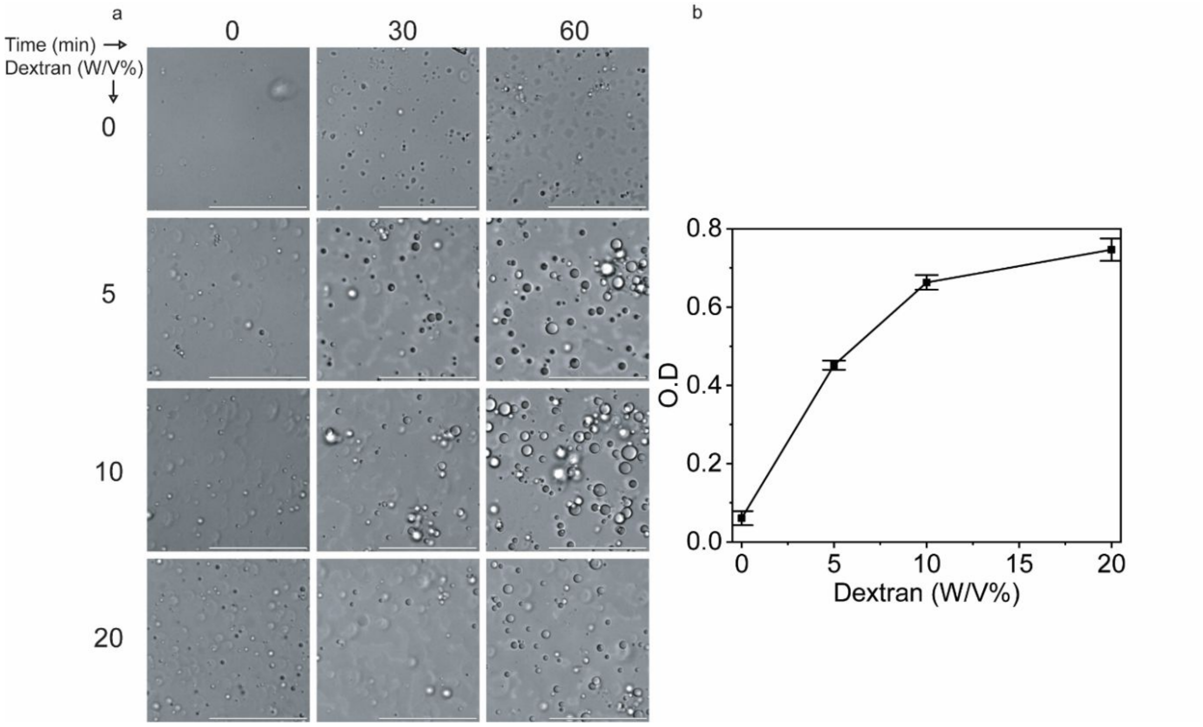

Figure S19. Molecular crowding increases DNA0-HNP LLPS propensity. a. Microscopy images of DNA0-HNP LLPS samples at increasing dextran concentrations, over one hour. **b**. Turbidity of LLPS samples at increasing dextran concentration, at t=0 min. Samples were prepared with 50 µM DNA0 and 740 µM HNP at 1:1 charge ratio, in Tris-HCl (10 mM, pH 8) containing 5 mM $MgCl_2$. Values represent averages of n=3-4 from 2 independent measurements. Scale bars = 50 µm.

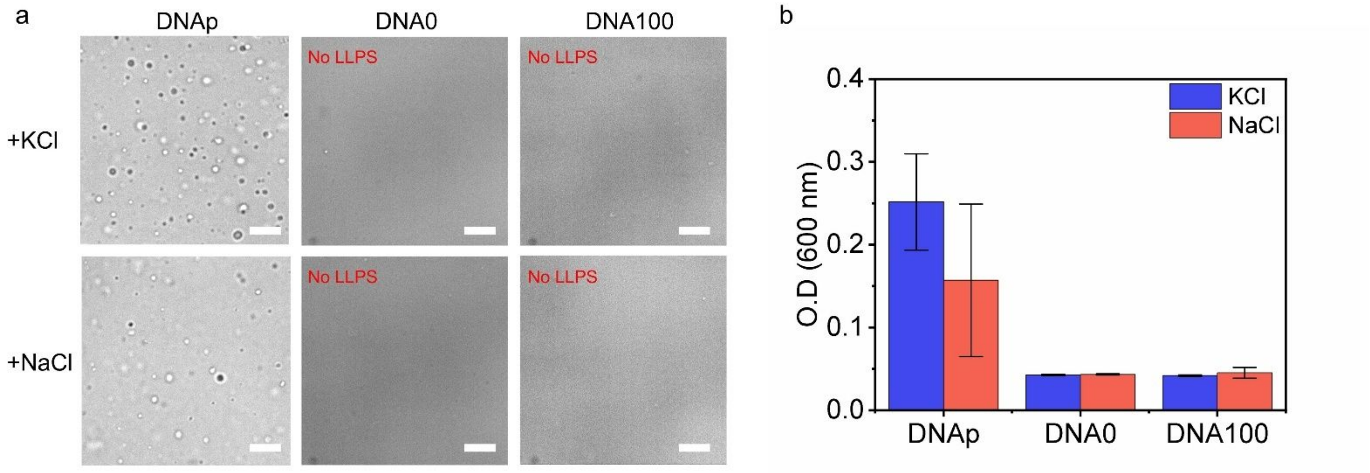

Figure S20. Physiological crowding and ionic strength modulate DNA-peptide LLPS. **a**. Representative microscopy images of DNA-peptide samples in the presence of dextran and salts, KCl (top) and NaCl (bottom). **b**. Turbidity measurements (mean ± S.D., n = 9 from three independent experiments). All samples contained 100 µM DNA and 1.48 mM HNP at a 1:1 charge ratio, with either KCl or NaCl (150 mM) in 20 mM HEPES (pH 7.5) and 20% (w/v) dextran. Scale bars = 10 µm.

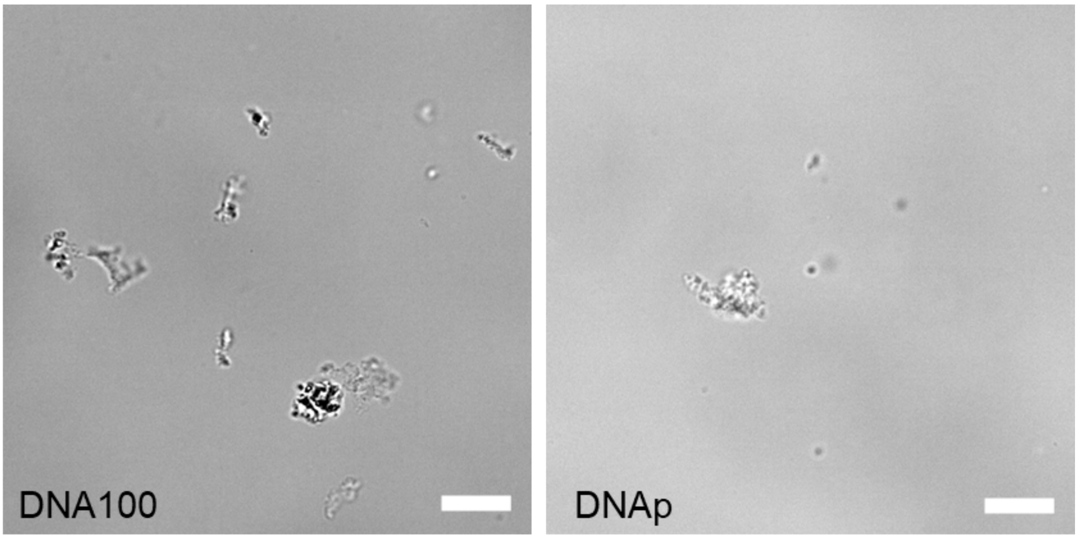

Figure S21. DNA aggregates. Bright field images of 100 µM DNA in Tris-HCl buffer pH 8 without $MgCl_2$ at room temp. Scale bars=10 µm.

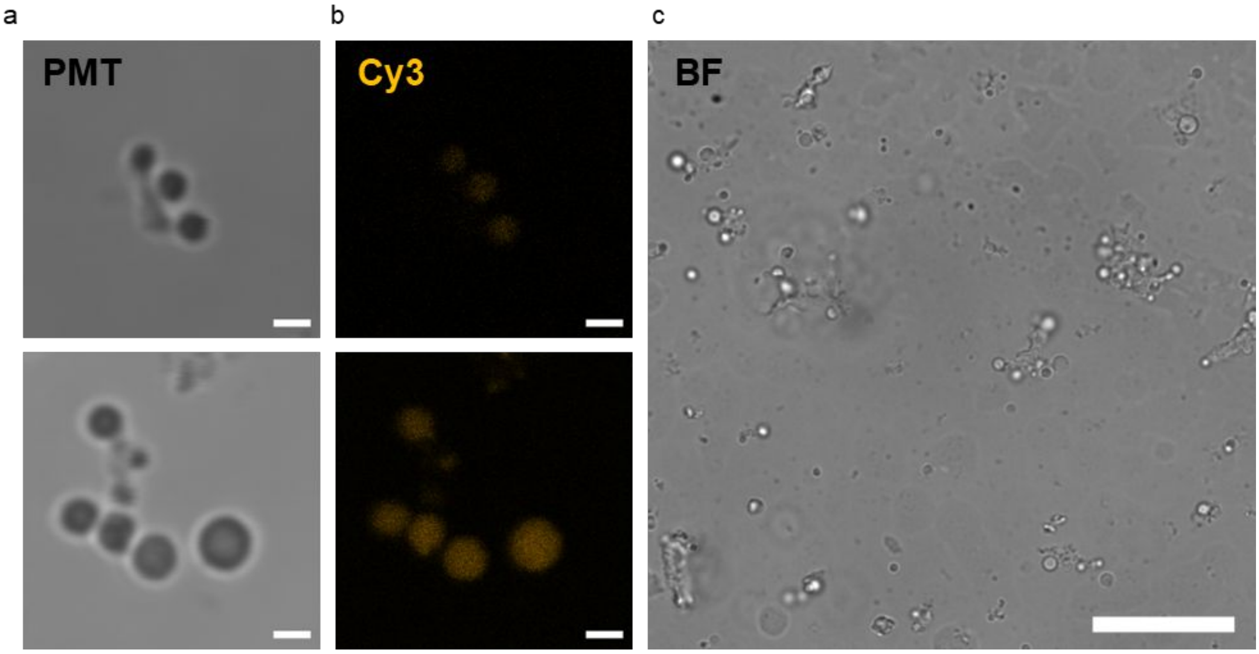

Figure S22. Droplet-aggregate structures of DNA100 with HNP. a-b. Confocal micrographs (**a-b**) showing transmitted light (**a**) and Cy3-DNA100 emission (**b**) or bright field micrograph (**c**) of DNA100/HNP LLPS samples (100 µM DNA100, 1.48 mM HNP) with 5 mM $MgCl_2$. Scale bar of (**a-b**) is 2 µm. Scale bar of (**c**) is 25 µm. Confocal images contain 50 nM Cy3-DNA100.

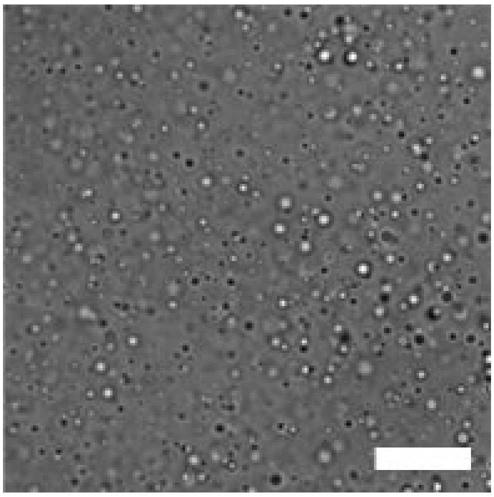

Figure S23. Transient LLPS of DNA100 and HNP without $MgCl_2$. Bright field micrograph of transient LLPS of DNA100/HNP without $MgCl_2$ and without pipetting. DNA100 concentration is 100 µM, DNA:peptide charge ratio is 1:1. Scale bar = 20 µm.

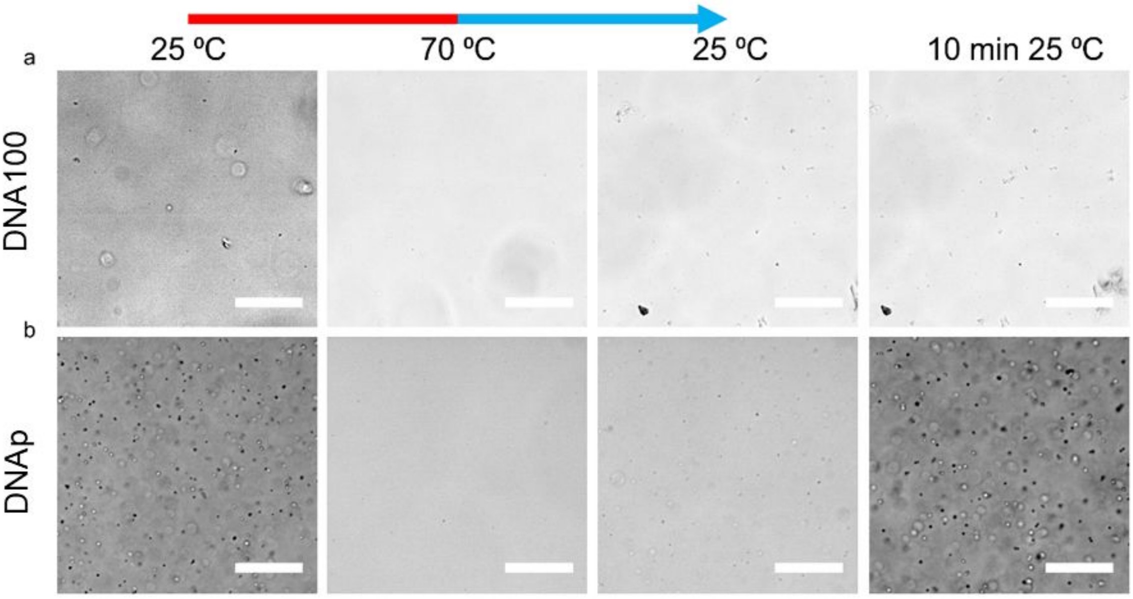

Figure S24. Effect of heating/cooling on DNA-HNP condensates. a-b. BF light microscopy images of DNA (100µM), HNP (1.48 mM) and $MgCl_2$ (10 mM) samples during heating/cooling of DNA100 (**a**) and DNAp (**b**). Scale bars = 50 µm.

Supplementary Figures Supporting the Computational Methods

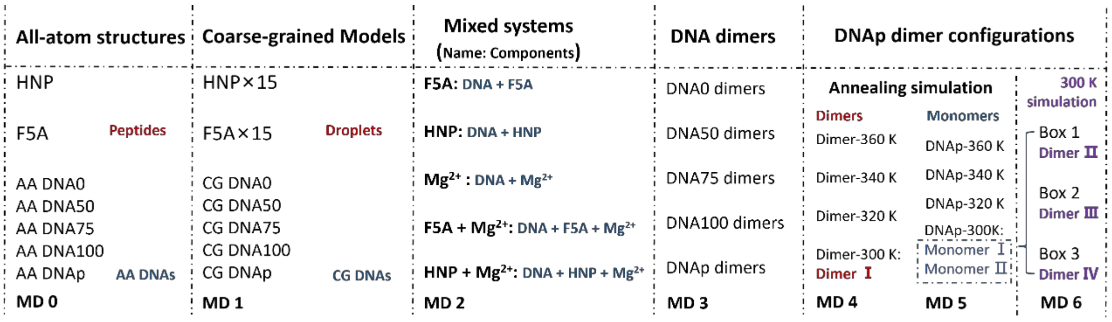

Figure S25. Workflow of CGMD simulations. The color code: red for peptides (HNP, F5A), blue for DNAs (DNA0, DNA50, DNA75, DNA100, DNAp), and mixed systems with Mg^{2+}.

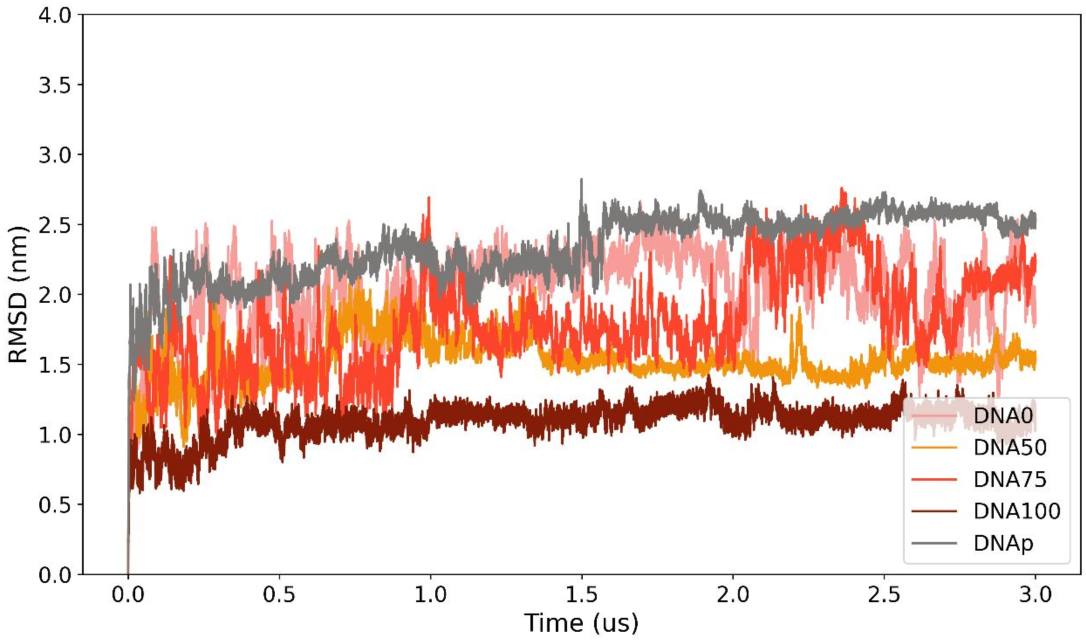

Figure S26. RMSD of DNA.

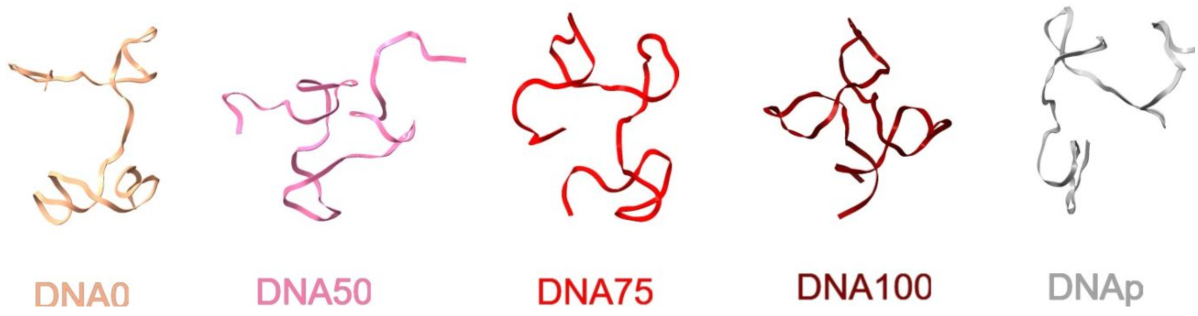

Figure S27. The initial all-atom DNA structure generated by modeling (see Computational Details).

.

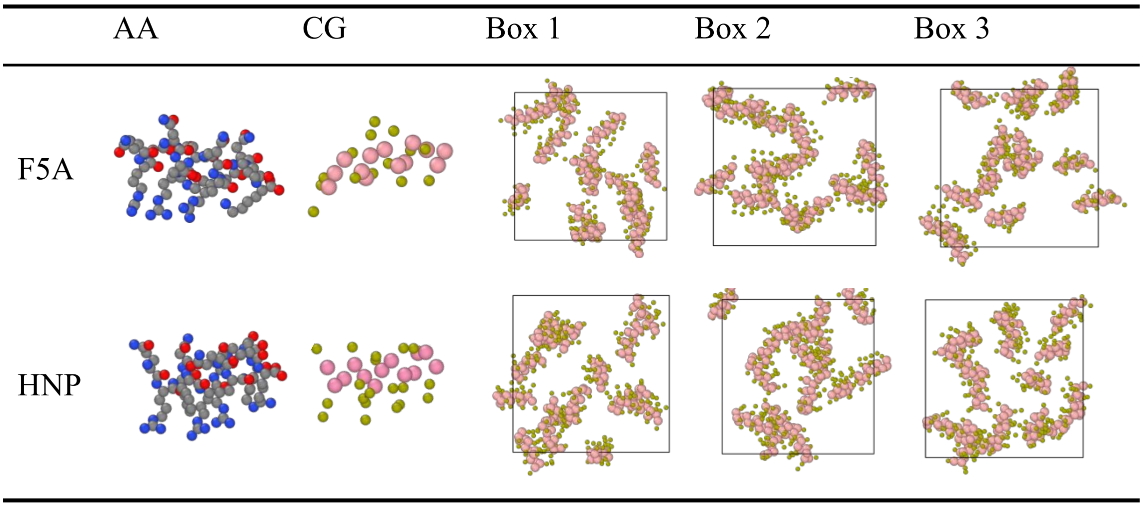

Figure S28. CGMD simulations of peptide droplets. From left to right: all-atom structure, CG structure of a single peptide, and three different boxes containing 15 CG peptides.

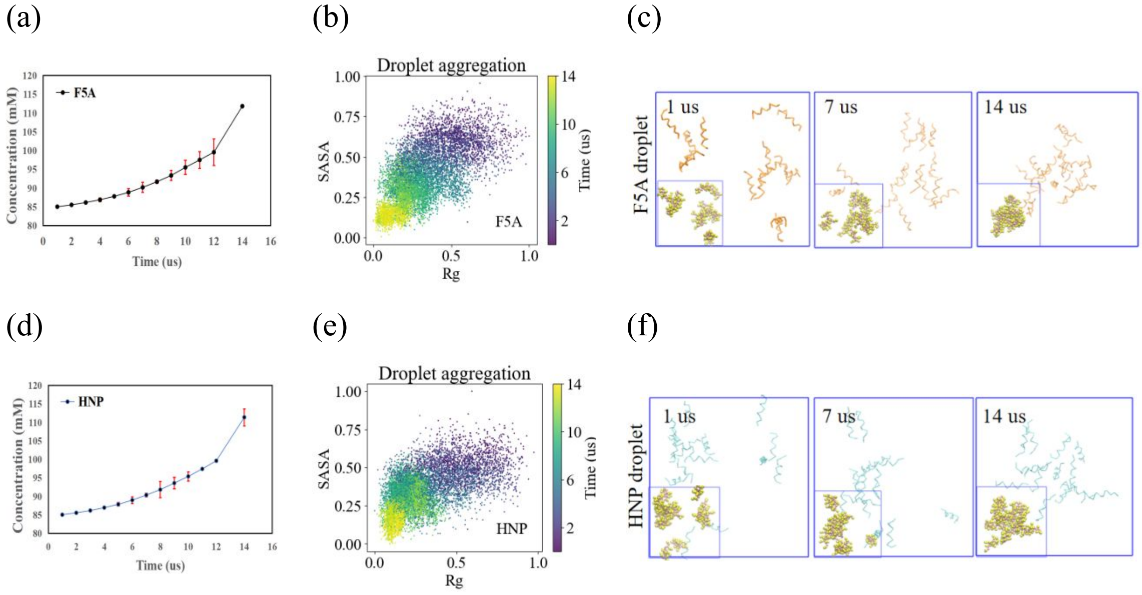

Figure S29. Aggregation of peptide droplets. (a-c) Aggregation of 15 F5A. (d-f) Aggregation of 15 HNP. (a) and (d) Changes in peptide concentration over time. (b) and (f) The aggregation process of droplets, where the normalized SASA and Rg were plotted, by mapping time values to a color gradient, the aggregation of the peptides can be clearly visualized, transitioning from purple to yellow, the degree of aggregation is highest near the point (0,0).

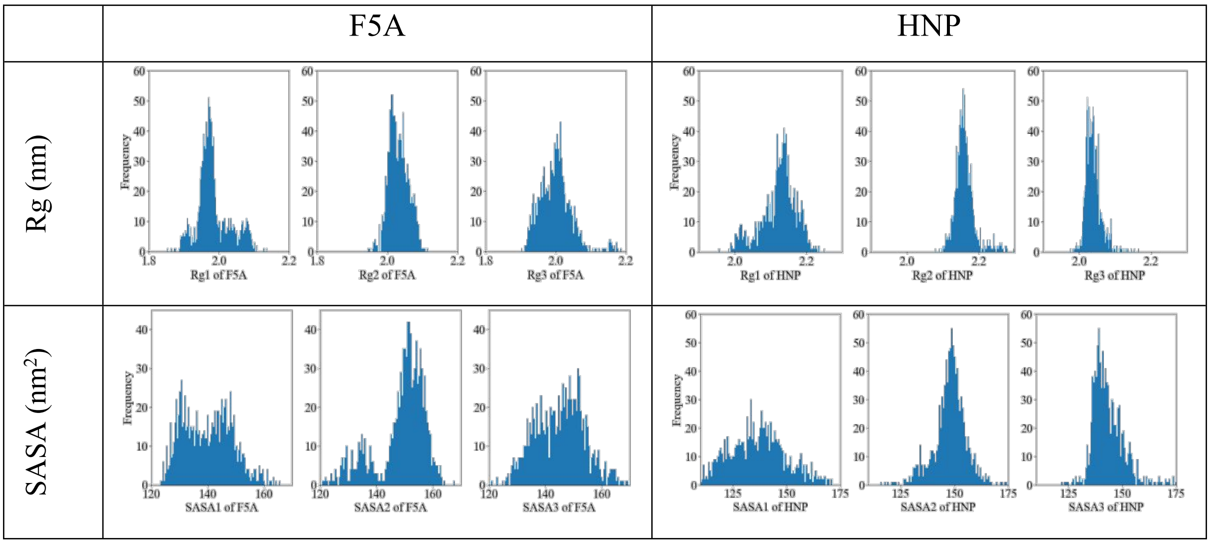

Figure S30. Rg and SASA distribution of peptide droplets. Three different distributions of Rg (namely Rg1, Rg2 and Rg3) and SASA (namely SASA1, SASA2 and SASA3) of F5A and HNP droplets.

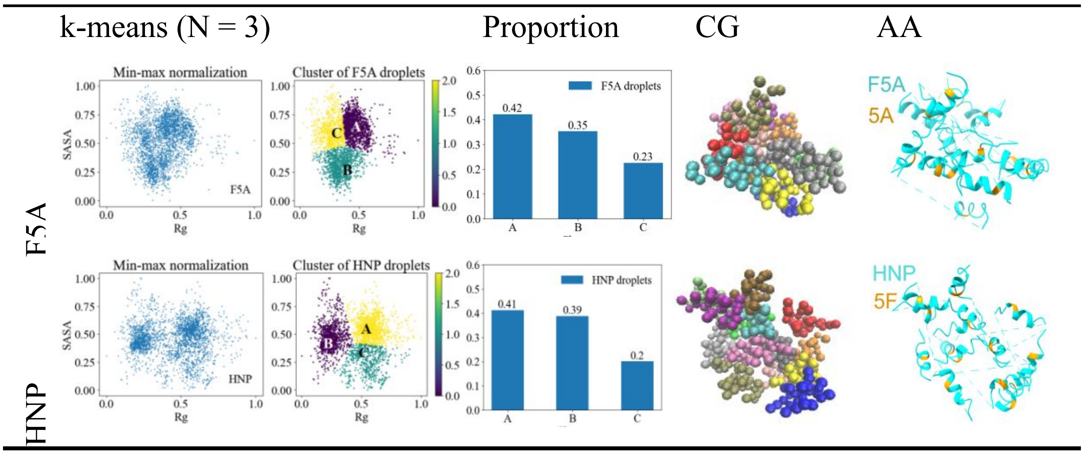

Figure S31. Clusters of droplet models. N is the number of clusters.

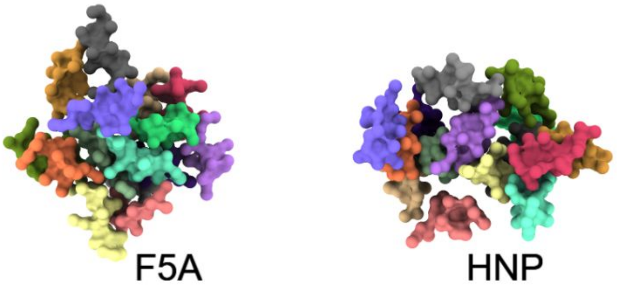

Figure S32. Coarse-grained peptide droplets models. CG F5A and HNP droplets model are built using k-means algorithm by clustering SASA and Rg.

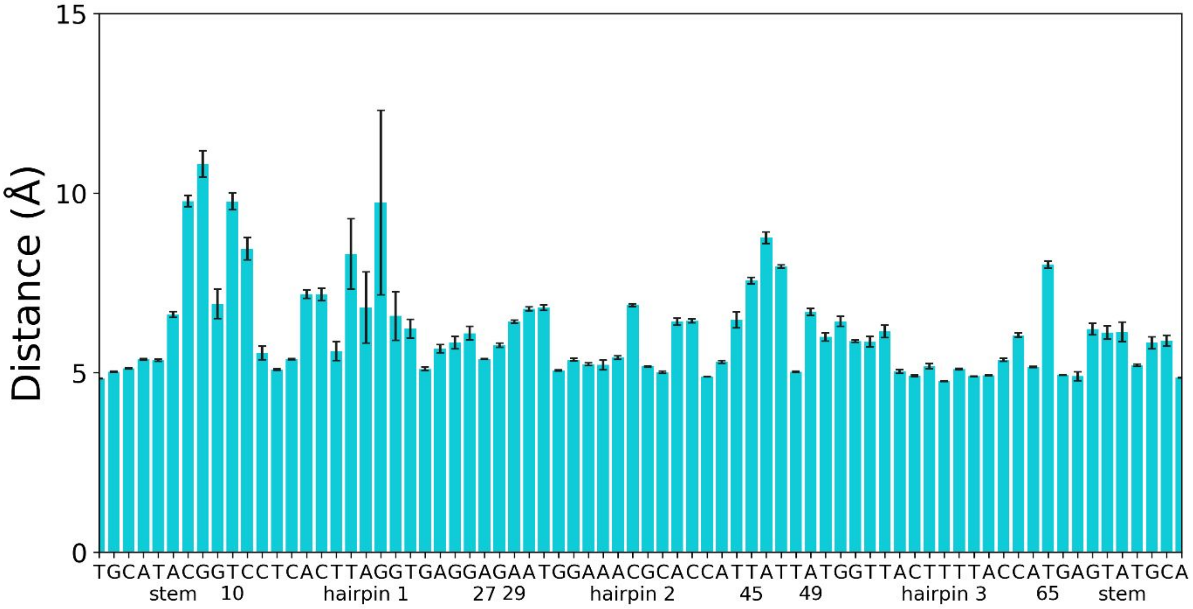

Figure S33. Average distance (Å) and variance between DNA100 and HNP obtained from CGMD simulations.

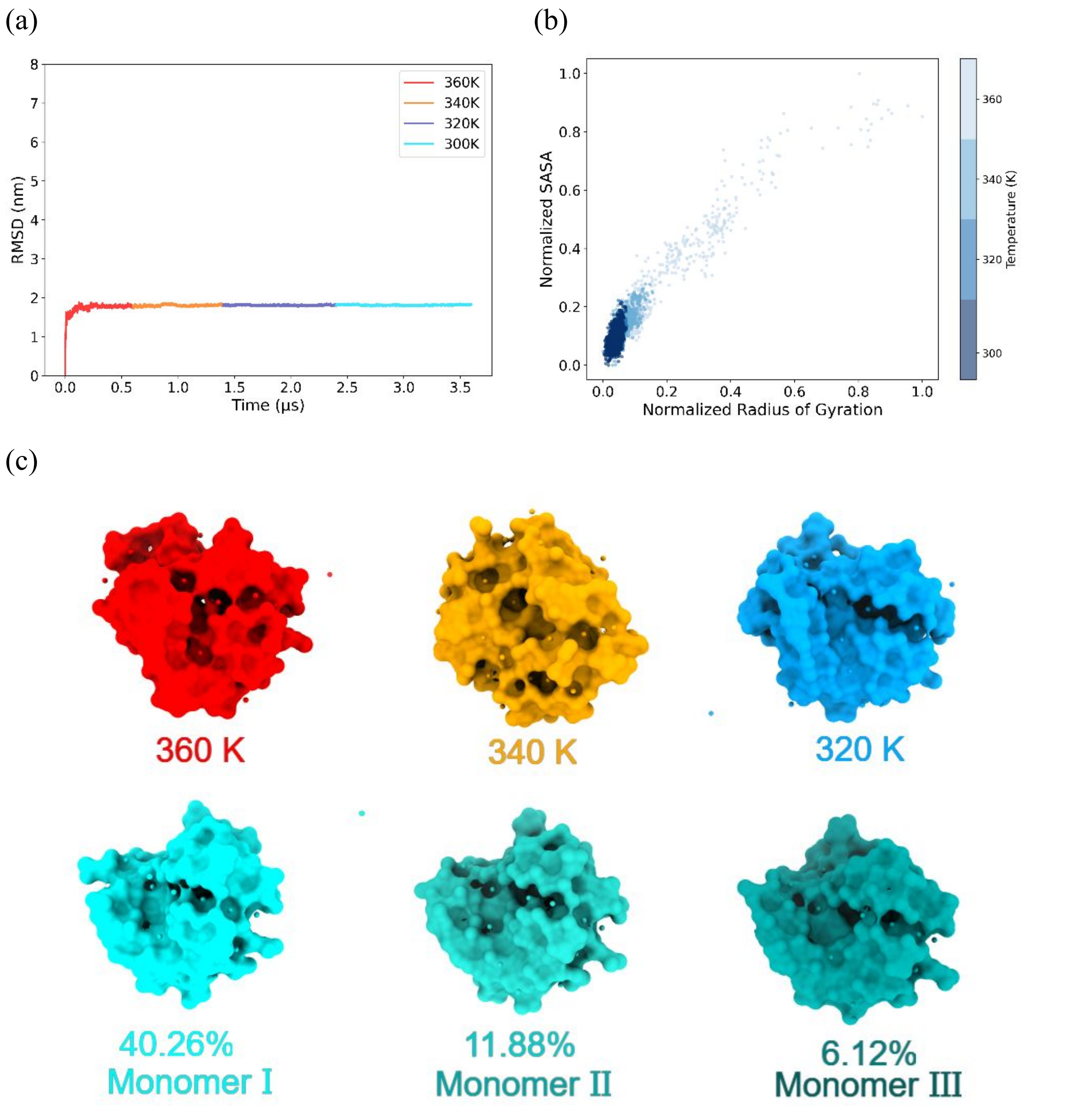

Figure S34. Structural ensemble of the DNAp monomer generated using the simulated annealing method. (a) RMSD over time at different temperatures (360 K, 340 K, 320 K and 300 K). (b) The formation process of DNAp monomers. (c) Trajectory clustering yields three representative structures.

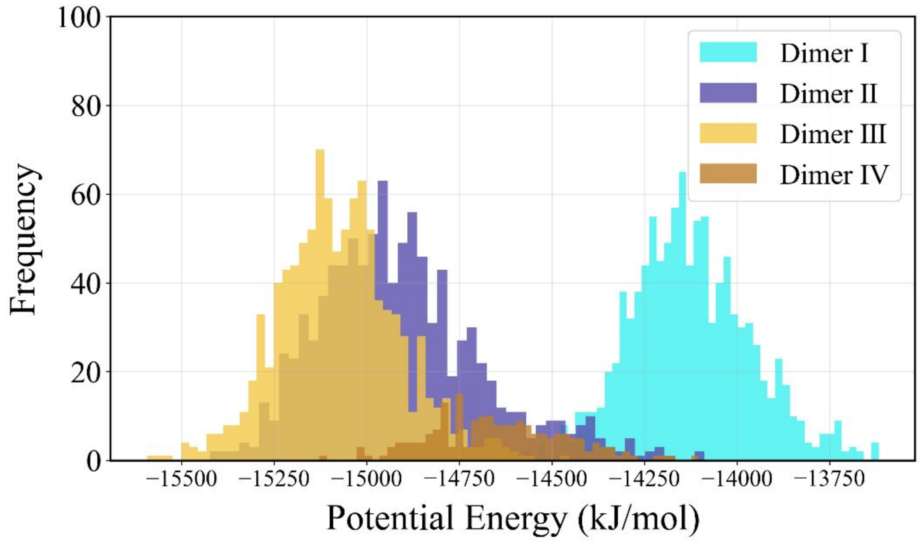

Figure S35. Potential energy distributions of four DNAp dimers.

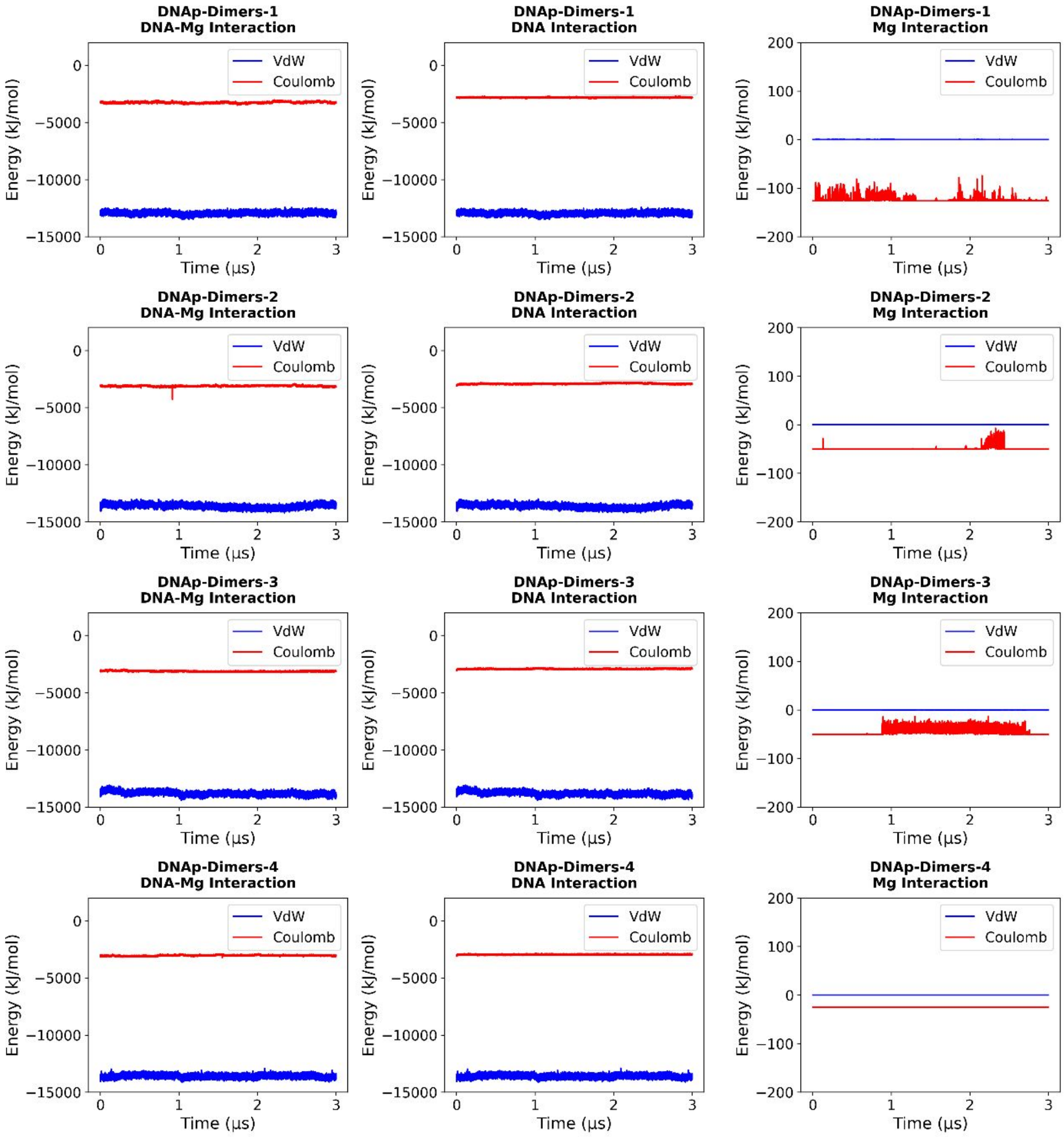

Figure S36. Non-bonded interaction energies from CGMD simulations. Shown are the interactions between DNA and Mg^{2+} ions (DNA-Mg), within the DNA dimer (DNA), and within clusters of Mg^{2+} ions (Mg) during the dimerization process.

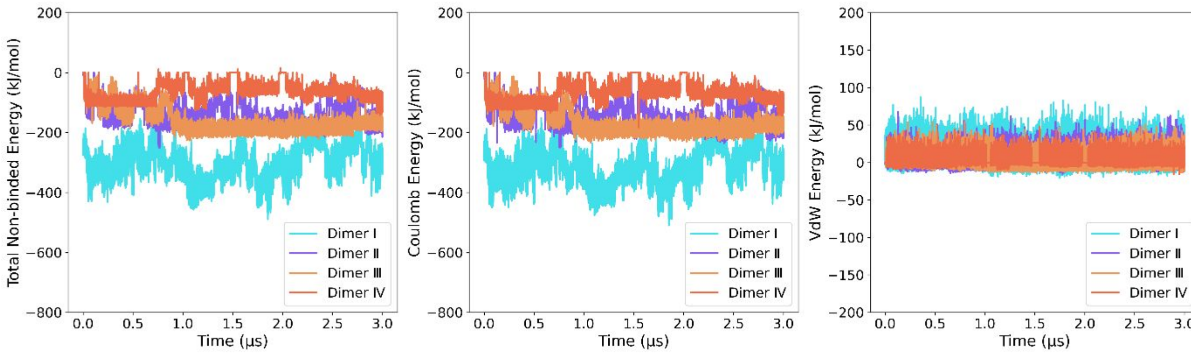

Figure S37. Non-bonded interaction energies of DNAp dimers over time.

Supplementary Tables

Table S1. Human tRNA and DNA sequences. tRNA original sequence and designed DNA sequences.

	Sequence
Human tRNA	GUUAAGACGGCAGAGCCCGGCAAUUGCGUAAAAUUUACAACUUUAUGGGCAGAGGUUCAAUUCCUCUUCUUAACA
Reversed transcribed tRNA	GTTAAGACGGCAGAGCCCGGCAATTGCGTAAAATTTACAACTTTATGGGCAGAGGTTCAATTCCTCTTCTTAACA
DNA0	TACTCGTTATTAAATCGACCGATCTGGTGGTATTCTTCAGGCAATAGGACATCTGGGCTTTGGAAAAATCACAG
DNA50	CATATTCGGCATTAGGGACGACTGTCTGAATGGAAACGCACCATTATTATGGTTACTTTTACCATGAGGGCTAA
DNA75	CATATTCGGTCCTCACTTAGGTGAGGAGAATGGAAACGCACCATTATTATGGTTACTTTTACCATGAGGGCTAA
DNA100	TGCATACGGTCCTCACTTAGGTGAGGAGAATGGAAACGCACCATTATTATGGTTACTTTTACCATGAGTATGCA
DNAp	TATCCAATCGACAATTCAGTCCAGTGTACGTACTTAATCATTTTAGGAGGTGGAAGGGGCTCGAAGTTATCTCA

**Table S2.** Predicted ΔG of DNAs. UNAfold outputs of predicted ΔG of most prominent structure of DNA100/DNAp/DNA0 at varying Mg^{2+} concentrations.						
Mg^{+2} (mM)	**DNA100**		**DNAp**		**DNA0**	
	ΔG (kcal mole^{-1})	T_m (C°)	ΔG (kcal mole^{-1})	T_m (C°)	ΔG (kcal mole^{-1})	T_m (C°)
0	-8.69	39.6	0.13	23.5	-1.22	44.6
1.5	-15.18	51	-1.19	29.6	-2.98	38.6
10	-17.46	55.3	-1.98	32.8	-3.82	42.7
40	-19.17	58.5	-2.58	35.2	-4.46	45.8

Table S3. The effect of $MgCl_2$ and HNP on DNA FRET efficiency and distance. d is the distance between Cy3 and Cy5, and SD represents standard deviation. The data was averaged from three parallel experiments with n=12.

	DNA0				DNAp				DNA100			
	E	SD	d (nm)	SD	E	SD	d (nm)	SD	E	SD	d (nm)	SD
DNA	0.10	0.05	7.50	0.68	0.09	0.08	8.32	2.23	0.36	0.24	5.76	0.98
+$MgCl_2$	0.11	0.05	7.29	0.53	0.12	0.08	7.46	0.93	0.80	0.02	4.04	0.10
+HNP	0.41	0.10	5.46	0.40	0.41	0.12	5.49	0.52	0.92	0.03	3.39	0.23
+$MgCl_2$ +HNP	0.44	0.10	5.34	0.37	0.45	0.09	5.29	0.33	0.92	0.02	3.34	0.2

**Table S4.** Rg and R_{max} derived from SAXS measurements (all data is in nm)				
DNA type	Rg (alone)	Rg (+HNP)	R_{max} (alone)	R_{max} (+HNP)
DNA100	1.8 ± 0.1	2.2 ± 0.5	5.55	8.56
DNA0	2.1 ± 0.1	1.95 ± 0.04	6.8	7.4
DNAp	2.34 ± 0.05	2.37 ± 0.08	7.6	8.0

Table S5. Setup details of CGMD simulations (T = 300 K)

System	Components	Water bead[*2]	Anti-frozen water bead[*3]	Na^+	Cl^-	Box (nm^3)	Simulation Time (μs)
MD 1	HNP×15 (droplet)	~2500	100	0	75	7×7×7	14 × 3
	F5A×15 (droplet)	~2500	100	0	75	7×7×7	14 × 3
MD 2	DNA only[*1]	45000	0	73	0	18×18×18	3
	DNA + F5A	45000	0	0	2	18×18×18	3
	DNA + HNP	45000	0	0	2	18×18×18	3
	DNA + Mg^{2+}	45000	0	0	1	18×18×18	3
	DNA + F5A + Mg^{2+}	45000	0	0	76	18×18×18	3
	DNA + HNP + Mg^{2+} [*4]	45000	0	0	76	18×18×18	3
MD 3	DNA dimer + Mg^{2+}	20500	0	30	32	14×14×14	3

[*1] Including DNA0, DNA50, DNA75, DNA100 or DNAp, respectively.
[*2] One water bead represents four H_2O molecules.
[*3] Anti-frozen water bead is an auxiliary particle that prevents system freezing.
[*4] For the DNA0 system, we further simulated the system of DNA0 + HNP + Mg^{2+} for 5 μs.

Table S6. Setup details of annealing CGMD simulations for DNAp dimers

System	Components	Water bead	Na^+	Cl^-	Box (nm^3)	T	Simulation time (μs)
MD 4	DNA dimer + Mg^{2+}	31500	30	32	16×16×16	360 K	0.6
						340 K	0.8
						320 K	1.0
						300 K	4.2
MD 5	DNA + Mg^{2+}	3437	5	6	8 × 8 × 8	360 K	0.6
						340 K	0.8
						320 K	1.0
						300 K	1.2
MD 6	DNA dimer + Mg^{2+}	20500	30	32	14×14×14	300 K	3.0 × 3

**Table S7**. Proportion of DNA clusters					
Type	Cluster 1	Cluster 2	Cluster 3	Number of clusters	Cutoff (nm)
DNA100	0.49	0.149	0.093	22	0.40
DNA75	0.47	0.240	0.205	8	1.10
DNA50	0.56	0.237	0.075	10	0.55
DNAp	0.49	0.196	0.134	13	0.55
DNA0	0.51	0.201	0.128	12	0.90

Table S8. $\bar{d}_{ii}$ (nm) and v_{ii} between *same type of bead* on 5' and 3' terminal of DNA

Systems		BB2*		BB3*		SC1*		SC2*		SC3*	
		$\bar{d}_{ii}$	v_{ii}	$\bar{d}_{ii}$	v_{ii}	$\bar{d}_{ii}$	v_{ii}	$\bar{d}_{ii}$	v_{ii}	$\bar{d}_{ii}$	v_{ii}
DNA100	DNA	1.501	0.281	1.416	0.306	1.163	0.363	1.074	0.410	1.039	0.415
	F5A	1.801	0.179	1.714	0.137	1.805	0.218	1.774	0.330	1.970	0.349
	HNP	1.754	0.122	1.711	0.106	1.756	0.137	2.008	0.168	1.907	0.206
	Mg^{2+}	1.348	0.149	1.352	0.152	1.158	0.190	1.192	0.309	1.038	0.252
	F5A + Mg^{2+}	1.597	0.145	1.539	0.097	1.254	0.124	1.084	0.223	1.041	0.182
	HNP + Mg^{2+}	1.312	0.097	1.146	0.074	1.333	0.065	1.466	0.067	1.599	0.077
DNAp	DNA	3.936	0.380	3.966	0.351	4.112	0.297	4.078	0.292	4.359	0.272
	F5A	2.809	0.294	2.735	0.283	2.677	0.312	2.559	0.419	2.741	0.402
	HNP	3.663	0.098	3.863	0.091	3.785	0.090	3.664	0.127	3.947	0.118
	Mg^{2+}	2.703	0.226	2.639	0.231	2.808	0.315	2.808	0.364	3.061	0.451
	F5A + Mg^{2+}	4.048	0.106	4.073	0.108	4.182	0.104	4.228	0.101	4.366	0.118
	HNP + Mg^{2+}	4.085	0.099	4.125	0.095	4.283	0.096	4.272	0.117	4.528	0.162

*In the Martini 2.1 coarse-grained model of DNA, BB2-3 represents the phosphate backbone, SC1-3 represents the base and pentose sugar.

Table S9. FRET efficiency ($\alpha = 0.1$) in CG simulation

System	DNA	F5A	HNP	Mg^{2+}	F5A + Mg^{2+}	HNP + Mg^{2+}
DNA100	0.233	0.261	0.397	0.418	0.538	0.989
DNAp	0.078	0.111	0.257	0.122	0.224	0.216
